# CiliaCarta: An integrated and validated compendium of ciliary genes

**DOI:** 10.1371/journal.pone.0216705

**Published:** 2019-05-16

**Authors:** Teunis J. P. van Dam, Julie Kennedy, Robin van der Lee, Erik de Vrieze, Kirsten A. Wunderlich, Suzanne Rix, Gerard W. Dougherty, Nils J. Lambacher, Chunmei Li, Victor L. Jensen, Michel R. Leroux, Rim Hjeij, Nicola Horn, Yves Texier, Yasmin Wissinger, Jeroen van Reeuwijk, Gabrielle Wheway, Barbara Knapp, Jan F. Scheel, Brunella Franco, Dorus A. Mans, Erwin van Wijk, François Képès, Gisela G. Slaats, Grischa Toedt, Hannie Kremer, Heymut Omran, Katarzyna Szymanska, Konstantinos Koutroumpas, Marius Ueffing, Thanh-Minh T. Nguyen, Stef J. F. Letteboer, Machteld M. Oud, Sylvia E. C. van Beersum, Miriam Schmidts, Philip L. Beales, Qianhao Lu, Rachel H. Giles, Radek Szklarczyk, Robert B. Russell, Toby J. Gibson, Colin A. Johnson, Oliver E. Blacque, Uwe Wolfrum, Karsten Boldt, Ronald Roepman, Victor Hernandez-Hernandez, Martijn A. Huynen

**Affiliations:** 1 Centre for Molecular and Biomolecular Informatics, Radboud University Medical Center, Nijmegen, the Netherlands; 2 Theoretical Biology and Bioinformatics, Science faculty, Utrecht University, Utrecht, the Netherlands; 3 School of Biomolecular and Biomedical Science, University College Dublin, Belfield, Dublin, Ireland; 4 Department of Otorhinolaryngology, Radboud University Medical Center, Nijmegen, the Netherlands; 5 Donders Institute for Brain, Cognition and Behaviour, Radboud University Medical Center, Nijmegen, the Netherlands; 6 Molecular Cell Biology, Institute of Molecular Physiology, Johannes Gutenberg University of Mainz, Mainz, Germany; 7 Great Ormond Street Institute of Child Health, University College London, London, United Kingdom; 8 Department of General Pediatrics, University Hospital Muenster, Muenster, Germany; 9 Department of Molecular Biology and Biochemistry and Centre for Cell Biology, Development and Disease, Simon Fraser University, Burnaby, British Columbia, Canada; 10 Medical Proteome Center, Institute for Ophthalmic Research, University of Tuebingen, Tuebingen, Germany; 11 Department of Human Genetics and Radboud Institute for Molecular Life Sciences, Radboud University Medical Center, Nijmegen, the Netherlands; 12 Section of Ophthalmology & Neurosciences, Leeds Institute of Molecular Medicine, University of Leeds, Leeds, United Kingdom; 13 Telethon Institute of Genetics and Medicine (TIGEM), Naples, Italy; 14 Medical Genetics, Department of Translational Medicine, Federico II University of Naples, Naples, Italy; 15 Institute of Systems and Synthetic Biology (iSSB), Genopole, CNRS, Univ. Evry, France; 16 Dept. Nephrology and Hypertension, Regenerative Medicine Center, University Medical Center Utrecht, Utrecht, the Netherlands; 17 Structural and Computational Biology Unit, European Molecular Biology Laboratory, Heidelberg, Germany; 18 Department of Human Genetics, Radboud University Medical Center, Nijmegen, the Netherlands; 19 Pediatric Genetics Division, Center for Pediatrics and Adolescent Medicine, University Hospital Freiburg, Freiburg, Germany; 20 Biochemie Zentrum Heidelberg (BZH), Heidelberg University, Heidelberg, Germany; 21 Bioquant/Cell Networks, Heidelberg, Germany; Justus Liebig Universitat Giessen, GERMANY

## Abstract

The cilium is an essential organelle at the surface of mammalian cells whose dysfunction causes a wide range of genetic diseases collectively called ciliopathies. The current rate at which new ciliopathy genes are identified suggests that many ciliary components remain undiscovered. We generated and rigorously analyzed genomic, proteomic, transcriptomic and evolutionary data and systematically integrated these using Bayesian statistics into a predictive score for ciliary function. This resulted in 285 candidate ciliary genes. We generated independent experimental evidence of ciliary associations for 24 out of 36 analyzed candidate proteins using multiple cell and animal model systems (mouse, zebrafish and nematode) and techniques. For example, we show that OSCP1, which has previously been implicated in two distinct non-ciliary processes, causes ciliogenic and ciliopathy-associated tissue phenotypes when depleted in zebrafish. The candidate list forms the basis of CiliaCarta, a comprehensive ciliary compendium covering 956 genes. The resource can be used to objectively prioritize candidate genes in whole exome or genome sequencing of ciliopathy patients and can be accessed at http://bioinformatics.bio.uu.nl/john/syscilia/ciliacarta/.

## Introduction

Cilia are microtubule-based organelles extending from the surface of most eukaryotic cells, serving critical functions in cell and fluid motility, as well as the transduction of a plethora of sensory and biochemical signals associated with developmental processes [1]. Disruption of cilia leads to a wide range of human disorders, known as ciliopathies, characterized by defects in many different tissues and organs leading to symptoms such as cystic kidneys, blindness, bone malformation, nervous system defects and obesity [2,3]. The cilium is a complex and highly organized structure, typically comprised of a ring of nine microtubule doublets extending from a centriole-derived basal body and enveloped by an extension of the plasma membrane. Importantly, cilia are compartmentalized structures, with a protein composition of the membrane and lumen differing significantly from that of the plasma membrane and cytoplasm [4,5]. Several hundred proteins are thought to be involved in the formation and function of ciliary structures and associated signaling and transport pathways. Between Gene Ontology (GO) [6] and the SYSCILIA Gold Standard (SCGS) [7] approximately 750 genes have already been associated with ciliary function. However, it is likely that many more ciliary proteins remain to be identified, as new genes for this organelle are still frequently uncovered—often in relation to genetic disorders. [2].

Although omics data sets provide a rich source of information to determine the proteins that constitute an organelle, they are inherently imperfect. It is well established that large scale approaches often miss key players; for example, proteomics fares better at finding intracellular versus extracellular or membrane proteins [8]. Combining data sets into a single resource that exploits their complementary nature is a logical step. The power of such an approach has been demonstrated for various cellular systems such as small RNA pathways [9], the innate antiviral response [10] and mitochondrial proteins in MitoCarta [11]. The latter is a compendium of mitochondrial proteins based on the integration of various types of genomics data that has been extensively used by the biomedical community [12].

Like for the mitochondrion, approaches that exploiting signals in genomics data to predict new genes have been applied to the cilium, e.g. the specific occurrence of genes in species with a cilium[13,14], the sharing of specific ciliogenic transcription factor binding sites such as the X-box motif [15] and the spatiotemporal co-expression of ciliary genes [16]. Furthermore, an extensive database, CilDB, contains data from individual genomics, transcriptomics, and proteomics approaches related to the cilium [17]. Although such databases are invaluable to researchers, it is not obvious how to handle the sometimes-conflicting information presented by independent data sources: for example, what does a negative result in one dataset and a positive result in another mean? A powerful solution lies in statistical and probabilistic integration of data sets. Multiple methods exist to combine genomics data, ranging from simply taking the genes that are reported by most data sources, to machine learning methods that take into account non-linear combinations of the data (reviewed in [18]). In this spectrum, naive Bayesian classifiers take a middle ground. They exploit the relative strengths of the various data sets while maintaining transparency of the integration. For each gene, the contribution of each data set to the prediction can be determined and new independent data sources can then simply be added to those already used to improve predictive value and coverage.

Here, we present CiliaCarta, a thorough wet-lab studies validated compendium of ciliary genes based on literature, annotation, and genome-wide Bayesian integration of a wide range of experimental data, that includes a recent large-scale protein-protein interaction data set specifically focused on ciliary proteins [19]. We extend the currently known set of cilia-related genes with 209 putative cilia-related genes, to a total of 956 genes. Based on the outcome of the probabilistic integration and the results from the experimental validations, we estimate the total size of the human ciliome to be approximately 1200 genes. Furthermore, we show that objective data integration using Bayesian probabilities is capable of overcoming biases based on literature. As proof-of-principle of our approach, we show that OSCP1, previously described as a solute carrier or tumor suppressor [20,21], is also a ciliary component, required for regulating vertebrate ciliogenesis and associated tissue homeostasis.

## Results

### Data collection and curation

We collected and constructed a total of six new data sets from proteomics, genomics, expression and evolutionary data and complemented these with two public data sets (Table 1). The data sets include three protein-protein interaction (PPI) data sets based on three methods: tandem-affinity purification and mass spectrometry (TAP-MS) [19], stable isotope labeling of amino acids in cell culture (SILAC), and yeast two-hybrid (Y2H) screens. The latter two data sets are published as part of this work and include 1666 proteins (1301 and 365, respectively) describing 4659 interactions (4160 and 499, respectively.) and an estimated positive predictive value for discovering new ciliary proteins of 0.28 and 0.54 respectively. Because the TAP-MS and SILAC data sets are based on a similar methodology and have a large bait overlap (14 out of 16 SILAC baits were used in the TAP-MS data set), we merged them into a single data set (Mass-spec based PPI) to avoid bias (see methods). Furthermore, we created three additional bioinformatics data sets: (i) a data set describing the presence or absence of conserved cilia-specific RFX and FOXJ1 transcription factor binding sites (TFBS) in the promoter regions of human genes, based on the 29 mammals project [22], (ii) a bioinformatics screen for genes that show co-expression with a set of known ciliary genes, and (iii) a comprehensive co-evolution data correlating gene presence-absence patterns with the presence of the cilium over a representative data set of eukaryotic species. Supplementing these six data sets are two published large-scale data sets of primary and motile cilia: (i) the Liu *et al*. study features a proteomics data set describing the protein content of sensory cilia derived from isolated murine photoreceptor cells [23] and was mapped to the human proteome via orthology as implemented in CilDB [17]; (ii) the Ross *et al*. study comprises of a dynamic gene expression data set describing the up-regulation of genes during ciliogenesis in a time series after shearing off cilia in human lung epithelial cells [24]. Although both data sets are not among the strongest predictors for ciliary function, they have a much higher coverage of the human proteome than other published data sets (S1 Fig). The eight data sets were selected to ensure independence of the evidence types and provide comprehensive coverage of molecular signatures for ciliary genes. Importantly, each individual data set contains a highly significant signal for the discovery of ciliary genes (Table 1). A full description on each data set is given in the methods section.

**Table 1:** Coverage and predictive power of the cilium data sets.

Dataset	# of genes in dataset	Coverage of genome	Coverage of Gold Standard	p-value
**TAP-MS**	4410	19.4%	64.9%	1.5E-67
**SILAC**	1397	6.2%	21.5%	3.8E-19
**Mass-spec based PPI (TAP-MS + SILAC)**	4799	21.1%	65.9%	2.0E-64
**Y2H PPI**	343	1.5%	9.2%	2.1E-14
**RFX/FOXJ1 TFBS**	2201	9.7%	29.4%	2.2E-22
**Cilia co-occurrence (DDP ≤ 9)**	1485	6.5%	30.5%	2.8E-37
**Expression screen (S ≥ 1.5)**	5448	24.0%	75.5%	2.4E-69
**Liu et al. 2007**	2085	9.2%	38.4%	1.4E-43
**Ross et al. 2007**	1204	5.3%	26.2%	2.4E-33

Coverage columns denote the fraction of the genome or SCGS that are identified by each approach. P-values indicate significant overrepresentation of the SCGS compared to random by Fisher’s exact test. Mass-spec based PPI represents the union of the TAP and SILAC data sets, resulting in the integration of five new and two published data sets.

### Bayesian integration of omics data sets provides gene-specific probabilities for cilia involvement

**Fig 1:**
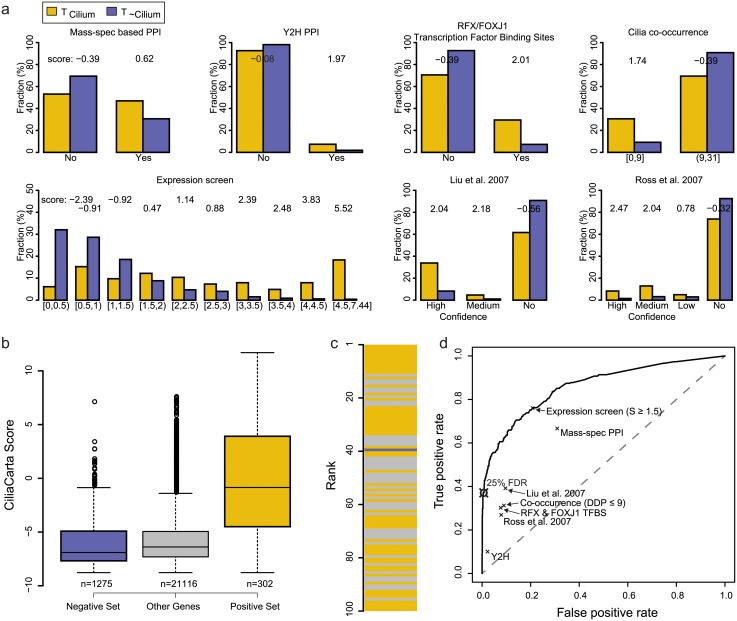
Data sets and performance of the Bayesian classifier for predicting ciliary genes. A) For each data set the fraction of positive (T_Cilium_) and negative gene sets (T_~Cilium_) and the log-likelihood scores are displayed per sub-category. B) Distributions of the integrated CiliaCarta scores for the negative set, the positive set, and the remaining unassigned genes. The positive set has significantly higher scores than the negative set (p-value: 2.8e-85 Mann-Whitney U test). C) Top 100 scoring genes. Known ciliary genes from the positive set are in yellow, genes from the negative set are in blue. High scoring genes in grey are prime candidate novel ciliary genes. D) Receiver-Operator Characteristics curve showing the performance of the Bayesian classifier as a function of the CiliaCarta Score and the performance of the individual data sets.

By combining the complementary sources of evidence for cilium function, we obtain a data-driven, high-confidence compendium of ciliary genes. To integrate the data sets in a probabilistic manner we assessed their ability to predict ciliary genes using the SCGS, a manually curated set of known ciliary genes (the ‘positive’ set) [7], and a set of genes whose proteins show non-ciliary subcellular localization and are most likely not involved in ciliary function (the ‘negative’ set; see methods). We divided each data set into appropriate sub-categories that reflect increasing propensities to report ciliary genes (Fig 1A). Then, for each data category we calculated the likelihood ratios of predicting ciliary genes versus predicting non-ciliary genes using Bayes’ theorem. The final log-summed likelihood ratio, which includes the prior expected probability to observe a ciliary gene in the genome, we call the ‘CiliaCarta Score’. This score represents the likelihood for a gene to be ciliary, based on all the data sets considered (Bayesian scores for all genes can be found in S1 Table). A practical interpretation of the log-summed likelihood ratio is that a positive score of 1 means that a gene is twice more likely to be ciliary then non-ciliary. Whilst a score of -1 means that a gene is twice more likely to be non-ciliary then ciliary. The integrated score readily distinguishes between the positive and negative set (Fig 1B, p-value: 2.8e-85 Mann-Whitney U test). A ten-fold cross-validation demonstrates that the results are robust, with an area under the curve of 0.86 (S2 Fig). The top-ranking genes are strongly enriched for known ciliary genes (Fig 1C). Note that known ciliary genes which score negatively should not be considered as potential non-ciliary, but rather should be considered to be poorly represented by the experiments that were integrated. However, the Bayesian classifier outperforms any individual data set, while achieving genome-wide coverage (Fig 1D).

### Experiments validate ciliary function for 67% of selected candidate genes

In order to validate the quality of our approach we performed a series of experimental tests to assess newly predicted candidate genes for ciliary localizations and/or function. We set our inclusion threshold at a False Discovery Rate of 25% (cFDR, corrected for the prior expectation to observe a cilium gene; see methods). 404 genes fall within this threshold, 285 of which were not in the training sets and thus constitute novel candidate ciliary genes. Eight genes from the negative set (*HSPH1*, *CALM3*, *COL21A1*, *CALM1*, *PTGES3*, *HSPD1*, *COL28A1*, and *PPOX*) occur in the top 404 genes. Literature searches reveal no previous connection of these genes to the cilium. Some high scoring non-ciliary genes are expected due to stochasticity in the underlying experimental data, although it is possible that a few of these may still have a ciliary function. The FDR for the 285 candidate genes is 33% when excluding genes from the training sets (see methods). From the candidates not previously known to have a ciliary association, we selected a total of 36 genes, spread evenly across the top predictions, for experimental validation (Table 2). The gene selection was performed as unbiased as possible, restricted by orthology (zebrafish, *Caenorhabditis elegans*) and the resources available to the participating labs. We performed validations in human, mouse, zebrafish and *C*. *elegans* (roundworms) by applying six distinct approaches to determine ciliary localization and function.

**Table 2:** Genes selected for validation and the validation outcomes.

		Phenotype based			Localisation based					
Gene Rank	Gene Symbol	Roaming (C. elegans)	Dyefilling (C. elegans)	Zebrafish morpho-linos	hTERT-RPE1 eCFP fusion	Lung epithelial cells (human)	hTERT-RPE1 cells	Retina cross-sections (mouse)	Ciliary phen./loc.?	Published (PubMed IDs)
38	C20orf26				Ax. & BB				yes	
50	EML1	++	++						no	
52	RIBC2					Ax.	BB	CC, BB, AC, PCM	yes	24424412
72	ARMC3					Ax.	NCL	CC, BB, PCR, PS	yes	26923438
80	SYNE1	++	++						no	28625779
88	EFHC2	++	++						no	
96	CFAP20				Ax. & BB				yes	
116	MAGI2	-	+						yes	24608321
117	SRGAP3	++	+/++	increased cilia length in kv, dilated phronephric duct					yes	26104135
119	FAM65B	++	++						no	
141	CCDC113					Ax.	NCL	Rtlt, BB, AC, PCR	yes	25074808
170	NBEA	-	++						yes	
184	CYB5D1				Ax. & BB				yes	
186	C6orf165					BB	NCL	Cone specific. PIS, PCR, BB.	yes	
190	DMD	++	+						yes	
198	PPP5C	++	++						no	29426949
202	MYO5B	++	++						no	
218	RALGAPA1	++	++						no	
232	CCDC147				BB				yes	
243	C12orf10	++	++						no	
257	C15orf27				BB				yes	
264	PLCB4	-	++						yes	
274	ENAH	-	++						yes	
278	EFCAB7	-	++						yes	24582806
305	IQCA1				NCL				no	
306	HIPK1	-	++						yes	
319	TTC18			decreased cilia length in kv, decreased cilia number in kv, dilated phronephric duct					yes	17971504 (2008)
337	SLC22A4	++	++						no	
341	TSSC1	++	++						no	
347	IPO5				BB				yes	23914977
348	HSPAL1				BB				yes	
349	VPS35	-	++						yes	
359	SKP1	++	++						no	
379	TEKT1				BB				yes	24521320
395	RAB36			decreased cilia length in kv, decreased cilia number in kv, dilated phronephric duct					yes	
402	OSCP1	-	+						yes	

Empty cells means not tested. Eleven proteins have been published as ciliary proteins since the start of the validation experiments. Pubmed identifiers are provided in the final column. Ax.: axonemal, BB: basal body, NCL: non-ciliary localization, CC: connecting cilium, AC: adjacent centriole, PCM: periciliary membrane, PCR: periciliary region, PS: photoreceptor synapse, Rtlt: Rootlet, PIS: photoreceptor inner segment. Roaming: “++” normal, “-”defective (p<0.0001). Dye uptake: “++” normal, “+/++” slightly reduced uptake, “+” mild reduced dye uptake, “-”defective dye uptake.

#### Validation by phenotype

**Fig 2:**
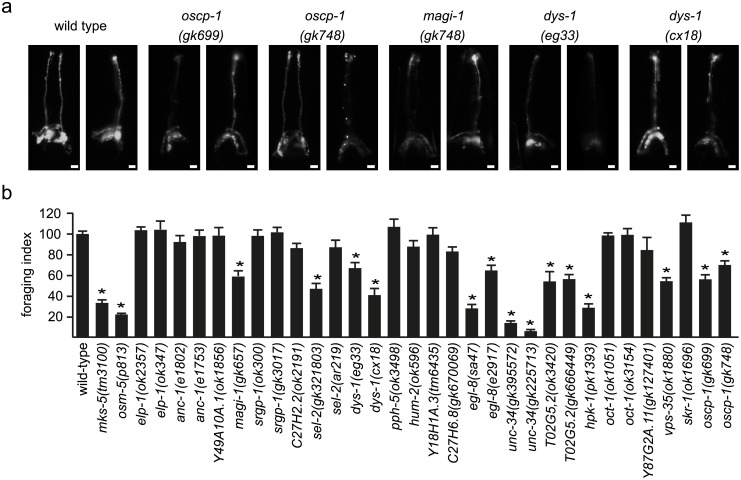
Validation by worm phenotype. A) *C*. *elegans* dye uptake assay. In wild type worms, DiI dye is taken up by 6 pairs of amphid (head) and one pair of phasmid (tail; not shown) neurons via their environmentally exposed sensory cilia. In the mutants shown, the amount of incorporated dye is modestly reduced, although most or all neurons still uptake the dye. The mks-5 mutant functions as control for ciliary disfunction. Scale bars: 20 μm. B) Single worm roaming assays. Bars represent mean ± S.E.M (n≥20) independent experiments), normalized to wild type control. * p<0.05 (unpaired t-test; vs. WT).

In the *C*. *elegans* hermaphrodite (959 cells), non-motile cilia extend from the dendritic endings of 60 sensory neurons. Most cilia are housed within the environmentally exposed cuticular pores, serving chemo-, osmo- and thermo-sensory roles [25]. 68 of our 285 candidate genes have one-to-one orthologs in *C*. *elegans*, and for 21 of these genes, viable alleles were available from the *Caenorhabditis* Genetics Center (University of Minnesota, USA) (S2 Table). These alleles are all nonsense or deletion mutations and predicted to severely disrupt gene function. For each available mutant, we employed dye-filling assays to indirectly assess the integrity of a subset of cilia (six amphid pairs and one phasmid pair), and foraging (roaming for food) and osmotic avoidance assays to investigate cilia-related sensory behaviors [26,27]. Nine mutants display roaming defects, with three of these mutants also showing a mild defect in dye uptake (Fig 2A and 2B, S3 Fig, and S2 Table). None of the mutants showed an abnormal osmotic avoidance response (S2 Table). Thus, 43% of assayed genes display associated mutant phenotypes consistent with a cilia-related role.

**Fig 3:**
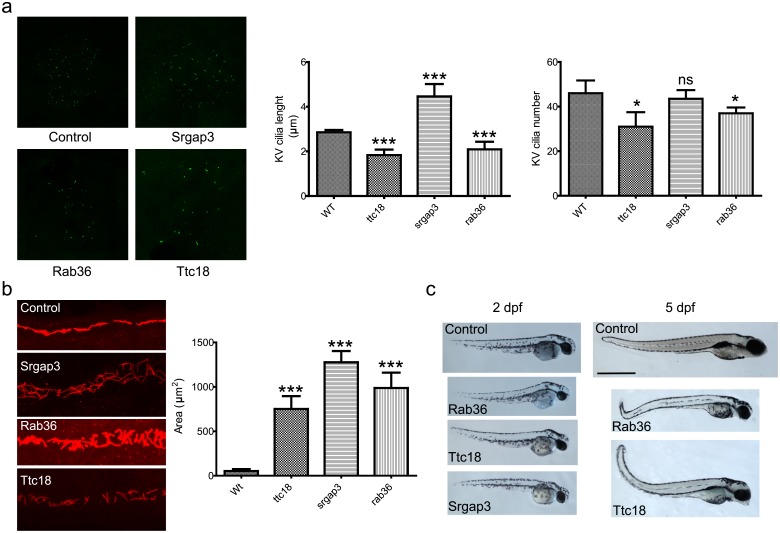
Validation by zebrafish phenotype. A) Cilia length and number in zebrafish Kupffer’s vesicles. The length and number of cilia in ttc18 and rab36 morphants are significantly reduced (p<0.001 and p<0.05 resp.). Cilia in srgap3 morphants are elongated (p<0.001) but the number of cilia is normal. Bars represent mean ± S.E.M. B) Pronephric ducts in 24 hpf morphants. Cilia are stained with antibodies against acetylated alpha tubulin. The pronephric ducts are significantly enlarged for all three morphants compared to wild type (p<0.001). Bars represent mean ± S.E.M. C) Whole embryo phenotype 2 days post fertilization (dpf) and 5 dpf zebrafish control and morphant embryos. All morphants exhibit the body curvature that is characteristic for cilia dysfunction. Note that in our screening we did not manage to obtain surviving srgap3 morphants past 3 dpf.

Three conserved candidate genes, *srgap3*, *ttc18* and *rab36*, were investigated in zebrafish for cilia-related phenotypes after knockdown with morpholinos. The development of cilia in the Kupffer’s vesicle was examined (Fig 3A). *rab36* and *ttc18* morphants show significantly reduced cilia length and number in the Kupffer’s vesicle (p<0.001 and p<0.05 respectively), while the *srgap3* morphant shows an increased cilia length (p<0.001), but no effect on cilia number. Pronephric ducts are several orders of magnitude larger in all three morphants during the pharyngula period (24 hpf) compared to wild type (Fig 3B, p-value < 0.001). Furthermore, all three morphants exhibit body-axis defects associated with cilium dysfunction [28,29] (Fig 3C).

Although the zebrafish morphant and *C*. *elegans* mutant allele data is consistent with ciliary functions for the associated genes, a few of these genes could orchestrate the observed phenotypes via non-ciliary roles or could be off-target effects (as is documented for morpholinos in zebrafish [30]). However, for two out of the three genes tested with zebrafish morpholinos (sr3gap and ttc18) a ciliary function has been established by others [31,32]. The high prevalence of cilia-related phenotypes in assayed mutants (e.g., roaming disruption [27,33–35]), implies a low (albeit not negligible) false negative rate and provides quantitative support for substantial enrichment of ciliary genes in CiliaCarta.

#### Validation by subcellular localization

**Fig 4:**
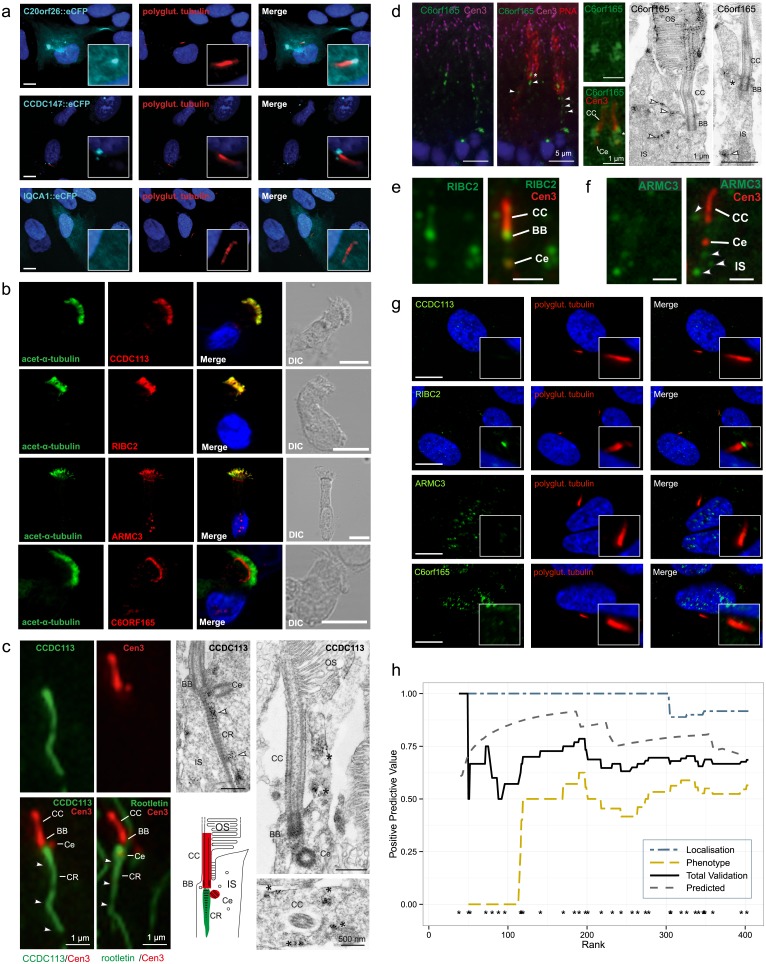
Validation by ciliary localization. A) Fluorescence microscopy of eCFP fused to C20orf26, CCDC147 and IQCA1 in hTERT-RPE1 cells. Acetylated alpha tubulin (red) is used to mark the axoneme. DAPI (blue) staining is used to mark the cell nucleus. IQCA1 does not appear to co-localize with acetylated alpha tubulin. B) Localization of CCDC113, RIBC2, ARMC3 and C6orf165 (red) compared with acetylated alpha tubulin (green) in human lung epithelial cells. C) Localization of CCDC113 in the primary sensory cilium of mature mouse photoreceptor cells. On the left: indirect 2-color immunofluorescence of CCDC113 (green) and centrin-3 (Cen3, red), a marker protein for the connecting cilium (CC), the basal body (BB) and the adjacent centriole (Ce), and of the ciliary rootlet (CR) marker rootletin (green) and Cen3 (red) indicates the localization of CCDC113 at the ciliary base and the CR projecting into the inner segment (IS). On the right: immunoelectron microscopy of CCDC113 confirms the localization of CCDC113 at the CR (arrowheads) and demonstrates accumulation of CCDC113 in the periciliary region of photoreceptor cells (asterisks). D) On the left: localization of C6orf165 in the primary sensory cilium of mature mouse cone photoreceptor cells. Indirect double immunofluorescence of C6orf165 (green) and Cen3 (magenta) in combination with the counterstaining with fluorescent PNA (red) revealed the localization of C6orf165 at the BB and the Ce of cone photoreceptors and a punctate staining in the IS (arrowheads). At the center: higher magnification of the double immunofluorescence of C6orf165 (green) and Cen3 (red). On the right: immunoelectron microscopy of the ciliary region of photoreceptors confirmed the ciliary and periciliary localization (asterisks) of C6orf165, but also demonstrated its presence in outer segments (OS) of cones. E) Double immunofluorescence of RIBC2 (green) and Cen3 (red) of a photoreceptor cilium showed localization of RIBC2 throughout the connecting cilium (CC) and the adjacent centriole (Ce) as seen by co-localization with the ciliary marker Cen3. F) Double immunofluorescence of ARMC3 (green) and Cen3 (red) of a photoreceptor cilium revealed the absence of ARMC3 from the CC (counterstained for Cen3) but a punctate staining in the periciliary region of the photoreceptor IS (arrowheads). G) Localization of CCDC113, RIBC2, ARM3 and C6orf165 compared with polyglutamylated tubulin in hTERT-RPE1 cells. H) Positive predictive value (PPV) of the Bayesian classifier based on the experimental validation outcomes plotted against CiliaCarta gene rank. The PPV of the combined validation converges to 0.67, which equals the predicted PPV (0.67, given 0.33 FDR). The asterisks (*) above the x-axis denote the ranks of the candidate genes and proteins tested for ciliary function or localization.

Subcellular localization studies were performed for a total of nine proteins in ciliated human hTERT-RPE1 cultured cells using eCFP-tagged overexpression constructs (Table 2, S4 Fig). We found that eight proteins localized to the basal body and/or the ciliary axoneme. To account for possible localization artifacts, two representative photos are taken per eCFP fusion protein, containing at least one ciliated cell from the same slide. Cells transfected for c15orf22::eCFP and c16orf80::eCFP ciliated only when expression of the eCFP fusion protein was low. Fig 4A shows representative examples, with C20orf26 and CCDC147 localizing to the basal body, whilst IQCA1 was not enriched at the cilium.

Four proteins (RIBC2, ARMC3, CCDC113 and C6orf165) were investigated for ciliary distribution of the endogenous protein by immunofluorescence microscopy. RIBC2, ARMC3 and CCDC113 co-localize with acetylated alpha tubulin, a marker for the ciliary axoneme in human respiratory cells, while C6orf165 specifically accumulates to the ciliary base (Fig 4B). We also tested these four proteins in other model systems to investigate if their ciliary localization is model-specific. Location was validated in murine retinal sections by immunofluorescence and at the ultrastructural level via immunogold electron microscopy (Fig 4C–4F). All four proteins associated with the sensory cilia of photoreceptor cells. However, in hTERT-RPE1 cells only RIBC2 showed localization at the basal body suggesting that ciliary targeting can be cell type specific (Fig 4G). Concurrent to our efforts other labs recently identified ciliary involvement for RIBC2 [36], ARMC3 [37] and CCDC113 [38].

#### Overall validation performance

Combining the results of all the validation experiments, we observed a ciliary localization or putative cilium-associated phenotype for 24 of the 36 candidates tested (Table 2), i.e. a positive predictive value (PPV) of 0.67. There appears to be a notable performance difference between the localization- and phenotype-based validation assays (PPV of 0.92 and 0.56 respectively, Fig 4H). This difference is likely attributable to the fact that knockdown of a ciliary gene does not necessarily lead to a detectable phenotype. The overall observed experimental PPV corresponds with the theoretical PPV of 67%, which corresponds to the FDR of 33% calculated for the candidate genes from the integrated CiliaCarta score (expected validation rate: 24.1 out of 36, p(x = 24 given 33% FDR) = 0.15, hypergeometric test, Fig 4H). The tested candidate genes have not been fully characterized, and thus it is possible that some might be false positives. However, even if we assume the lowest PPV of 40%, the experimental validation rate is significantly higher than expected by chance, based on the estimated prior distribution of ciliary versus non-ciliary genes in the human genome (expected 1.8 out of 36, p = 3.34e-23 for PPV = 67% and p = 7.64e-10 for PPV = 40%; hypergeometric test).

The experimental verification of our candidate genes therefore validates our Bayesian classifier.

### OSCP1, a novel ciliary protein

Unbiased integration of large-scale genomics data can give rise to apparent inconsistencies with previous literature reports. For instance, organic solute carrier partner 1 (OSCP1, or oxidored-nitro domain-containing protein 1, NOR1), scores high on our CiliaCarta list (ranked 402), despite reports of varied functions not obviously consistent with a ciliary role, such as regulation of inflammation, apoptosis, proliferation and tumor suppression [21,39,40]. OSCP1 was first implicated as a tumor suppressor in nasopharyngeal cancer [21] and later found to modulate transport rates of organic solutes over the plasma membrane in rodents [20,41]. These two facets of OSCP1 function have never been connected to each other in literature. Further research indicated a role for OSCP1 in regulation of inflammation and apoptosis [39], and that it is specifically expressed in mouse testes [42]. The availability of a substantial set of literature on OSCP1 without any indication of ciliary involvement, would have suggested that OSCP1 could have been a false positive in our Bayesian method. However, our *C*. *elegans* phenotype screen suggested that *C*. *elegans oscp-1* (R10F2.5) mutant alleles might possess modest sensory cilia defects (Fig 2A and 2B). Therefore, we targeted OSCP1 for more detailed investigation of OSCP1 in *C*. *elegans*, zebrafish and mammalian cells.

#### OSCP1 locates at the base and axoneme of the cilium in human, mouse and C. elegans cells

**Fig 5:**
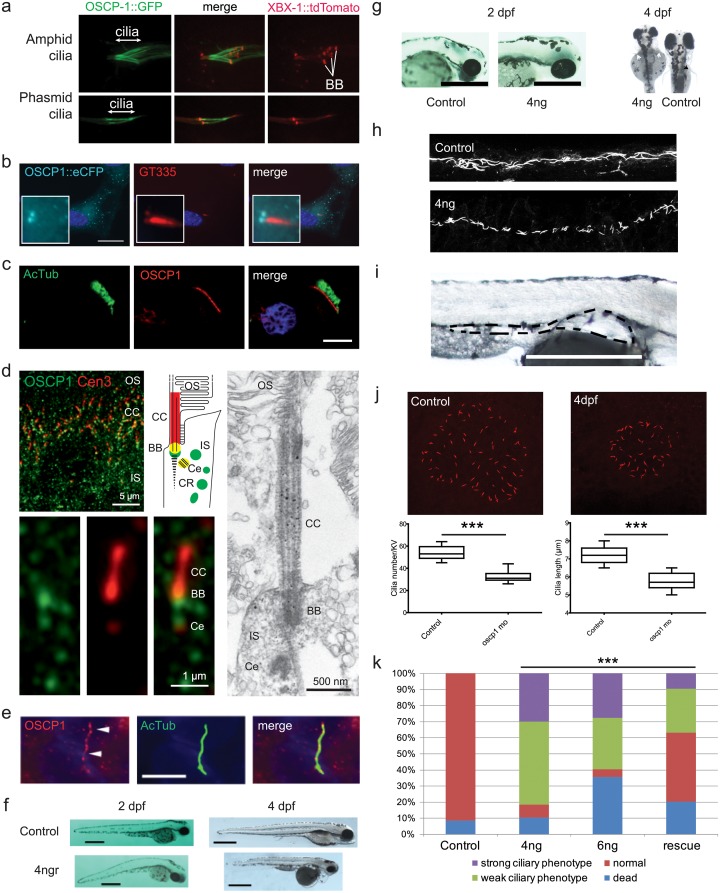
OSCP1 localizes to the cilium and regulates ciliary function in vivo. A) GFP-tagged OSCP-1 driven by its endogenous promoter is specifically expressed in ciliated sensory neurons in *C*. *elegans*, and the GFP-fusion protein is concentrated along the length of the cilium. Shown are fluorescent images of OSCP-1::GFP and XBX-1::tdTomato (ciliary marker) localization in amphid and phasmid cilia. Basal bodies (bb) and cilia are indicated. B) OSCP1::eCFP localization in hTERT-RPE1 cells. OSCP1 localizes to the basal body and daughter centriole as well as in the cytosol in a punctate manner. C) OSCP1 localization in human respiratory cells (red) co-stained with acetylated tubulin (green). OSCP1 localizes to the cytosol, but specifically to the base of the ciliated crown of these multi-ciliated cells. D) Indirect high magnification immunofluorescence of OSCP1 (green) and centrin-3 (Cen3, red), a marker protein for the connecting cilium (CC), the basal body (BB) and the adjacent centriole (Ce), in the photoreceptor cilium region of an adult mouse. Immunoelectron microscopy of CCDC113 confirms the localization of OSCP1 at the base of the cilium. The schematic represents a zoom of the ciliary region of a photoreceptor stained for OSCP1 and Cen3 in the according colors. IS, inner segment; OS, outer segment. E) Immunostaining of serum-starved murine IMCD3 cells with OSCP1 antibodies. OSCP1 is expressed in a punctate pattern along the axoneme. Acetylated tubulin and γ-tubulin (green), OSCP1 (Proteintech, 12598-1-AP, red) and nuclei (blue). Scale bar 5 μm. F) Zebrafish embryos injected with 4 ng of oscp1 splice morpholino 2 and 4 days post-fertilization (dpf). The characteristic ciliary phenotype with a curved body, small eyes and melanocyte migration defects at 2dpf. 4 dpf morphants display obvious pronephric cysts, small eyes, heart edemas, small heads and short bodies. Scale bar 500 μm. G) Left panel: details of the head of 2 dpf zebrafish embryos showing small eyes and small head in the oscp1 morphants. Scale bar 500 μm. Right panel: dorsal view of 4 dpf zebrafish embryos. Left embryo is a 4dpf oscp1 morphant. Right embryo is a control. Scale bar 200 μm. Note the small eyes, melanocyte migration defects and small fin buds (white arrows) in the oscp1 morphants compared with the control fin buds (black arrows). H) Immunofluorescence staining of pronephric cilia at 24 hpf (acetylated α-tubulin). 4 ng oscp1 morphants display shortened and disorganized cilia in the medial portion of the pronephric ducts. I) Detail of a pronephric cyst (outlined) in a 4 dpf zebrafish morphant. Scale bar 500 μm. J) Oscp1 morphants Kupffer’s vesicle cilia staining. Oscp1 morphants show smaller Kupffer’s vesicles with reduced cilia number per Kupffer’s vesicle (56 controls vs. 33 oscp1 morphants) and shorter cilia (controls; 7.1 μm vs oscp1 morphants; 5.7 μm. Significance was determined by t-test p-value<0.01. K) Dose dependent phenotype of oscp1 morphants. After injecting 4 ng of oscp1 morpholino the percentage of embryos with a weak phenotype is 51% and embryos with a strong phenotype is 28%. Those percentages change when 6 ng of morpholino are used with 31% of embryos showing with a weak phenotype and 27% with a strong phenotype, (strong and weak phenotypes described in S7 Fig). The number of dead embryos increases when 6ng of oscp1 morpholino are used (38%) compared with 4 ng of oscp1 morpholino (10%). We injected zebrafish embryos at one cell stage with 4 ng of oscp1 morpholino and 100 pg of human OSCP1 mRNA. The rescue increased the normal phenotype percentage from 8% to 43% and decreased the weak phenotype from 51% to 27% and the strong phenotype from 28% to 9.5%. Significance was determined by χ2 test, p<0.0001.

In *C*. *elegans*, expression of GFP-tagged OSCP-1, driven by its endogenous promoter, is exclusive to ciliated sensory neurons (the only ciliated cells in the nematode), indicating a high likelihood of a cilium-associated function (Fig 5A and S5 Fig). Analysis of the subcellular distribution pattern confirmed this by showing that OSCP-1::GFP localizes specifically to ciliary axonemes, including the ciliary base (Fig 5A and S5 Fig). In human hTERT-RPE1 cells, OSCP1::eCFP localizes at the basal body and daughter centriole of ciliated cells and at the centrioles of non-ciliated cells, indicating a basal body/centriole role for OSCP1 in this epithelial cell type (Fig 5B). In some cells an additional clear punctate localization in the cytoplasm can also be observed.

To assess the localization of the endogenous OSCP1 protein in various mammalian cells, we used commercially available antibodies from three suppliers (Proteintech, Atlas and Biorbyt). In human multi-ciliated respiratory cells, OSCP1 shows an increased concentration at the base of the cilia (Fig 5C). In murine photoreceptors OSCP1 is localized at the inner segments (Fig 5D), where it is particularly abundant at the base of the connecting cilium (the equivalent of the transition zone in primary cilia), and at low concentration at the adjacent centriole. Immunogold electron microscopy analysis also shows OSCP1 at the basal body, occasionally at the connecting cilium and sporadically within the inner segment (Fig 5D). Serum-starved IMCD3 cells (murine inner medullary collecting duct cells) showed ubiquitous punctate cytoplasmic staining with increased signal at the basal body and along the ciliary axoneme (Fig 5E and S6 Fig). In ATDC5 cells (murine pre-chondrocyte cells) OSCP1 is mainly localized to the ciliary axonemes, although the ciliary base signal is less apparent (S6 Fig).

Together, these exhaustive subcellular localization analyses place OSCP1 at ciliary structures, including the ciliary base and axoneme, although there are some subtle differences between cell types. We also frequently observed extensive non-ciliary signals for OSCP1, consistent with published reports for OSCP1 localizations at other organelles (ER, Golgi, Mitochondria) and within the cytosol[43,44].

#### OSCP1 is required for cilium formation in multiple zebrafish tissues, but dispensable in C. elegans sensory neurons

To investigate possible ciliogenesis roles for OSCP1, we first examined cilia in zebrafish morphants depleted for *oscp1* in fertilized eggs. In embryos at 2 days post fertilization (dpf) developmental phenotypes were observed that are often associated with ciliary defects: a curved body axis, small eyes and melanocyte migration defects (Fig 5F). Likewise, the 4 dpf morphants presented with pronephric cysts, small eyes, heart edema, small heads and short bodies (Fig 5F and 5G). The cilia in the medial portion of the pronephric ducts of *oscp1* morphants were shortened and disorganized (Fig 5H and 5I), and in the Kupffer's vesicle cilium length and number was decreased compared to control injected larvae (Fig 5J). Co-injecting human *OSCP1* mRNA together with the morpholino partially rescued the phenotype, indicating that is specific for loss of *oscp1* function (Fig 5K and S7 Fig). Therefore, in zebrafish, *oscp1* is required for cilium formation and associated functions in many tissue types and organs.

In contrast, analysis of the *oscp-1* (*gk699*) null allele in *C*. *elegans* revealed that OSCP-1 is not required for cilium formation. Using fluorescence reporters and transmission electron microscopy, the amphid and phasmid channel cilia appeared to be intact and full length (S5 and S9 Figs). In addition, *oscp-1* does not appear to be functionally associated with the transition zone at the ciliary base; disruption of this prominent ciliary domain (‘gate’) in the *mks-5* mutant [45] does not affect OSCP-1::GFP distribution, and loss of *oscp-1* itself does not influence the localization of several transition zone proteins (S5 Fig). Thus, OSCP1 is differentially required for cilium formation in worms and zebrafish. These distinctions could reflect redundancy of OSCP1 function with another ciliary protein in the nematode, but not in zebrafish, or differences in cell type requirements (nematode sensory neurons versus zebrafish epithelial cells). Clearly, based on its restricted expression in ciliated cells and localization to the ciliary axoneme, *C*. *elegans* OSCP-1 is serving a ciliary function, although the specifics of this role remain to be elucidated.

## Discussion

With the current interest in cilia biology, it is certain that many new genes will be implicated in ciliary function for several years to come. With the advent of systems biology and the need to understand the cilium as a whole the research community requires an inventory of genes and proteins involved in ciliary structure and function. The obvious sources for such an inventory, GO [6] and the SCGS [7], currently only cover 747 human genes (677 in GO as of March 20th 2018, 302 for the SCGS). By applying a naive Bayesian integration of heterogeneous large-scale ciliary data sets we have expanded this set by 28% to 956 human genes, adding 209 putative genes to the known cilium gene repertoire (S8 Fig). We put forward these putative ciliary genes, together with the SCGS and the GO annotated genes, as the “CiliaCarta”, a compendium of ciliary components with an estimated FDR of 7% (S3 Table). This community resource can be used to facilitate the discovery of novel cilium biology and to identify the genetic causes of cilia related genetic disease. CiliaCarta therefore has a different purpose than the SCGS; it serves as a tool for discovery of new genes, rather than as a reference of known cilium genes. Due to the additive nature of the naïve Bayesian classifier, researchers can easily apply the CiliaCarta score to their own results by sorting their candidate genes by the CiliaCarta score or by applying the mathematics detailed in S1 File.

Additional studies will help validate the newly found cilia gene candidates in CiliaCarta and will likely uncover other candidates. Estimates of the ciliary proteome range from one to two thousand proteins depending on the techniques used and on the types of cilia and the species studied [23,46]. In addition to obtaining the CiliaCarta list of ciliary genes, our Bayesian analysis allows us an objective estimate of the total number of ciliary genes. Using the posterior probabilities and the outcome from the validation experiments we estimate the size of the ciliome to be approximately 1200 genes (Methods).

Predicting the genes responsible for an organelle structure or function poses the question of where we draw the boundary between that organelle and the rest of the cell. For example, does the basal body in its entirety belong to the cilium or are some components to be considered exclusive to the centrioles? Furthermore, one can argue that in the case of the cilium, proteins that are not part of the cilium, but play a role in the transport of proteins to the ciliary base or regulation of cilium gene expression, can be regarded as components of the ‘ciliary system’. In practice, what we regard here as a ciliary component depends on the definition within the SCGS, which is used to weigh the data sets, and on our experimental validations. In both we have taken a rather inclusive approach by regarding genes whose disruption cause ciliary phenotypes as ciliary genes. This approach makes our predictions relevant to human disease. Indeed KIAA0753, which falls within our 25% cFDR list of cilium genes, was shown to interact with OFD1 and FOR20 at pericentriolar satellites and centrosomes, and gives rise to oral-facial-digital syndrome [47], a phenotype associated with disruption in the ciliary transition zone and the basal body.

Data integration through objective quantification of the predictive value of individual large-scale data sets enables the finding of new functions and associations, minimizing bias from previous studies. As a case in point we report here that OSCP1, not previously implicated in ciliary functions based on the existing published literature, is validated as a ciliary protein in four species as determined by multiple independent experimental methods. Re-evaluation of previous experimental evidence on OSCP1 function does not exclude ciliary involvement. Nevertheless, the cilium contains several specific ion-channels in its membrane [48] and the organelle has been implicated to play a role in the development of cancer [49–52]. Therefore, connecting OSCP1 to the cilium might provide the missing link to connect previously observed effects of OSCP1 on organic solute in-/efflux and its role in nasopharyngeal cancer. Our results therefore provide a cellular target and biomolecular framework to further unravel OSCP1 function.

Systematic integration of heterogeneous large-scale cilia data sets by employing Bayesian statistics combined with medium throughput experimental validation is a powerful approach to identify many new ciliary genes and provides a molecular definition of the cilium. The experimental observations of a potential ciliary role for selected high-confidence candidates, together with the results from the cross validation, indicates that the top tier of the Bayesian ranked human genome is highly enriched for ciliary genes. The genome-wide CiliaCarta Score and ranking, should therefore facilitate efficient and objective prioritization of candidate genes in order to discover novel ciliary genes and their functions.

## Materials and methods

### Ethics statement concerning human and animal subjects and tissue sampling

Analysis of human respiratory cells obtained by nasal brush biopsy is included in PCD-study protocols (2010-298-b-S; 2015-104-f-S) that were approved by the German Institutional Ethics Review Board from the University Hospital Muenster and University of Muenster (der Ärztekammer Westfalen-Lippe und der Medizinischen Fakultät der Westfälischen Wilhelms-Universität), in agreement with the principles of the Declaration of Helsinki. Healthy control subjects provided informed consent to analyze respiratory cells by immunofluorescence microscopy.

All the zebrafish work has been performed under the ASPA UK Home Office regulation (https://www.gov.uk/guidance/guidance-on-the-operation-of-the-animals-scientific-procedures-act-1986) within the Procedure Project License PPL number 70/7892. The adult zebrafish where maintained in compliance with the ASPA (Animals (Scientific Procedures) Act 1986) that regulates the use of animals in experiments and testing.

C57Bl/6J wild-type mice were kept on a 12 h light-dark cycle with unlimited access to food and water at the University’s Translational Animal Research Center (TARC) in Mainz, Germany. Mice were killed by cervical dislocation; the eyes were enucleated and immediately embedded and frozen. All procedures were in accordance with the guidelines set by the ARVO statement for the use of animals in Ophthalmic and Vision Research and the national and local laws on animal welfare (approved by the Kreisverwaltung Mainz-Bingen, Germany, with the animal breeding allowance number 41a/177-5865-§11 ZVTE).

### Data set collection and mapping

All data sets were mapped to the ENSEMBL human gene set version 71, release April 2013[53]. Resources using other identifiers (i.e. Entrez, Uniprot) were mapped to ENSEMBL gene IDs using ENSEMBL BioMART (version 71). Orthologs from non-human data sets were mapped using the ENSEMBL Compara ortholog catalogue from ENSEMBL 71, with exception of Sanger sequences from Y2H screens based on the Bovine cDNA library that were mapped to Bovine genome sequences by the BLAT tool [54] in the UCSC genome browser [55] and subsequently mapped to human orthologs using the 'non-cow RefSeq genes track' from this browser.

### DNA constructs

Bait protein selection was based on the association of proteins with ciliopathies (including mutant vertebrates showing ciliopathy features), involvement in IFT or part of our candidate list of ciliary proteins. Gateway-adapted cDNA constructs were obtained from the Ultimate ORF clone collection (Thermo Fisher Scientific) or generated by PCR from IMAGE clones (Source BioScience) or human marathon-ready cDNA (Clontech) as template and cloning using the Gateway cloning system (Thermo Fisher Scientific) according to the manufacturer’s procedures followed by sequence verification.

### Yeast two-hybrid system

A GAL4-based yeast two-hybrid system was used to screen for binary protein-protein interactions with proteins expressed from several different cDNA libraries as described previously [56]. Yeast two-hybrid constructs were generated according to the manufacturer’s instructions using the Gateway cloning technology (Thermo Fisher Scientific) by LR recombination of GAL4-BD Gateway destination vectors with sequence verified Gateway entry vectors containing the cDNA’s of selected bait proteins.

Constructs encoding full-length or fragments of bait proteins fused to a DNA-binding domain (GAL4-BD) were used as baits to screen human oligo-dT primed retinal, brain (Human Foetal Brain Poly A+ RNA, Clontech), kidney (Human Adult Kidney Poly A+ RNA, Clontech) or testis cDNA libraries, or a bovine random primed retinal cDNA library, fused to a GAL4 activation domain (GAL4-AD). The retina and testis two-hybrid libraries were constructed using HybriZAP-2.1 (Stratagene), the brain and kidney two-hybrid libraries were constructed using the “Make Your Own Mate & Plate Library System” (Clontech).

The yeast strains PJ96-4A and PJ96-4α (opposing mating types), which carry the *HIS3* (histidine), *ADE2* (adenine), *MEL1* (α-galactosidase), and *LacZ* (β-galactosidase) reporter genes, were used as hosts. Interactions were identified by reporter gene activation based on growth on selective media (*HIS3* and *ADE2* reporter genes), α-galactosidase colorimetric plate assays (*MEL1* reporter gene), and β-galactosidase colorimetric filter lift assays (*LacZ* reporter gene).

### Affinity purification of protein complexes using SILAC

#### DNA constructs and cell culture

Experiments were essentially performed as described before [57]. In short, N-terminally SF-TAP-tagged bait proteins that were obtained by LR recombination of TAP-destination vector with sequence verified Gateway entry vectors containing the cDNA’s of selected bait proteins using Gateway cloning technology (Thermo Fisher Scientific). HEK293T cells were seeded, grown overnight, and then transfected with SF-TAP-tagged bait protein constructs using Effectene (Qiagen) according to the manufacturer’s instructions. HEK293T cells were grown in SILAC cell culture medium as described [57].

#### Affinity purification of protein complexes

For one-step Strep purifications, SF-TAP–tagged proteins and associated protein complexes were purified essentially as described previously[57]. In short, SILAC labeled HEK293T cells, transiently expressing the SF-TAP tagged constructs were lysed in lysis buffer containing 0.5% Nonidet-P40, protease inhibitor cocktail (Roche), and phosphatase inhibitor cocktails II and III (Sigma-Aldrich) in TBS (30 mM Tris-HCl, pH 7.4, and 150 mM NaCl) for 20 minutes at 4°C. After sedimentation of nuclei at 10,000 g for 10 minutes, the protein concentration was determined using a standard Bradford assay. Equal protein amounts were used as input for the experiments to be compared. The lysates were then transferred to Strep-Tactin-Superflow beads (IBA) and incubated for 1 hour before the resin was washed 3 times with wash buffer (TBS containing 0.1% NP- 40 and phosphatase inhibitor cocktails II and III). The protein complexes were eluted by incubation for 10 minutes in Strep-elution buffer (IBA). The eluted samples were combined and concentrated using 10-kDa cutoff VivaSpin 500 centrifugal devices (Sartorius Stedim Biotech) and prefractionated using SDS-PAGE and in-gel tryptic cleavage as described elsewhere[58].

#### Quantitative mass spectrometry

After precipitation of the proteins by methanol-chloroform, a tryptic in-solution digestion was performed as described previously[59]. LC-MS/MS analysis was performed on a NanoRSLC3000 HPLC system (Dionex) coupled to a LTQ OrbitrapXL, respectively coupled to a LTQ Orbitrap Velos mass spectrometer (Thermo Fisher Scientific) by a nano spray ion source. Tryptic peptide mixtures were automatically injected and loaded at a flow rate of 6 μl/min in 98% buffer C (0.1% trifluoroacetic acid in HPLC-grade water) and 2% buffer B (80% acetonitrile and 0.08% formic acid in HPLC-grade water) onto a nanotrap column (75 μm i.d. × 2 cm, packed with Acclaim PepMap100 C18, 3 μm, 100 Å; Dionex). After 5 minutes, peptides were eluted and separated on the analytical column (75 μm i.d. × 25 cm, Acclaim PepMap RSLC C18, 2μm, 100 Å; Dionex) by a linear gradient from 2% to 35% of buffer B in buffer A (2% acetonitrile and 0.1% formic acid in HPLC-grade water) at a flow rate of 300 nl/min over 33 minutes for EPASIS samples, and over 80 minutes for SF-TAP samples. Remaining peptides were eluted by a short gradient from 35% to 95% buffer B in 5 minutes. The eluted peptides were analyzed by using a LTQ Orbitrap XL, or a LTQ OrbitrapVelos mass spectrometer. From the high-resolution mass spectrometry pre-scan with a mass range of 300–1,500, the 10 most intense peptide ions were selected for fragment analysis in the linear ion trap if they exceeded an intensity of at least 200 counts and if they were at least doubly charged. The normalized collision energy for collision-induced dissociation was set to a value of 35, and the resulting fragments were detected with normal resolution in the linear ion trap. The lock mass option was activated and set to a background signal with a mass of 445.12002[60]. Every ion selected for fragmentation was excluded for 20 seconds by dynamic exclusion.

For quantitative analysis, MS raw data were processed using the MaxQuant software [61] (version 1.5.0.3). Trypsin/P was set as cleaving enzyme. Cysteine carbamidomethylation was selected as fixed modification and both methionine oxidation and protein acetylation were allowed as variable modifications. Two missed cleavages per peptide were allowed. The peptide and protein false discovery rates were set to 1%. The initial mass tolerance for precursor ions was set to 6 ppm and the first search option was enabled with 10 ppm precursor mass tolerance. The fragment ion mass tolerance was set to 0.5 Da. The human subset of the human proteome reference set provided by SwissProt (Release 2012_01 534,242 entries) was used for peptide and protein identification. Contaminants like keratins were automatically detected by enabling the MaxQuant contaminant database search. A minimum number of 2 unique peptides with a minimum length of 7 amino acids needed to be detected to perform protein quantification. Only unique peptides were selected for quantification.

### Protein-protein interaction data processing

Based on three ciliary protein-protein interaction (PPI) data sets, we inferred proteins to "interact with ciliary components" as a proxy for being part of the cilium. First, we obtained protein complex purification data from a large-scale study on the identification of ciliary protein complexes by tandem-affinity purification coupled to mass spectrometry (TAP-MS) for 181 proteins known or predicted to be involved in ciliary functions [19]. Since integration of this data set into the CiliaCarta many pull-downs were repeated and reverse experiments included. As a result, our data set includes 539 found interactors that are not part of the now published final data set (4702 proteins, S2 Table from Boldt *et al*. [19]), and does not include 679 new interactors that have been identified since June 2013. The complete and current data is available at http://landscape.syscilia.org/ and IntAct [IM-25054]. Second, we obtained affinity purification data for 16 bait proteins from a more sensitive and quantitative approach using affinity purification combined with stable isotope labeling of amino acids in cell culture (SILAC). In total 1301 interactors were identified by SILAC in 57 experiments (S4 Table). Third, we also obtained direct protein-protein interaction data from several independent yeast two-hybrid (Y2H) screens against cDNA libraries derived from hTERT-RPE1 (retinal pigment epithelial) cells, as well as brain, kidney, retina and testis tissue. In total 69 Y2H screens were performed using 27 baits, identifying a total of 343 interacting proteins (S5 Table).

The SILAC and Y2H studies were focused on finding new interactors for selected ciliary proteins of interest and were not part of a systematic analysis. Parts of the resulting PPIs were published in previous studies (four out of 16 baits for SILAC [57,62,63] and nine out of 27 baits for Y2H [63–71]), however here we consider the entire PPI data sets. The complete data sets are publicly available in S4 and S5 Tables. Because the TAP-MS and SILAC data sets are based on similar methodology and have a large bait overlap (14 out of 16 SILAC baits were used in the TAP-MS data set), we merged them into a single data set (Mass-spec based PPI) with 4799 unique proteins identified to interact with 184 bait proteins.

The Y2H, SILAC and TAP-MS data were transformed to genome wide data sets by defining genes as 1 (“found”) when the gene product was found to interact with the baits, and as 0 (“not found”) when the gene product was not found to interact. Due to the large overlap in baits and the largely similar methods used in the TAP-MS and SILAC data sets we decided to combine the data sets in order to avoid counting the interacting proteins multiple times and thereby artificially overestimating their CiliaCarta Scores. The mass-spectrometry data sets and Y2H data sets were found to be sufficiently different to include them as separate data sets (positive set correlation is 0.13, S10 Fig).

### Expression screen data set

In expression screening [72] separate gene-expression data sets are weighted for their potential to predict new genes for a system by measuring, per data set, the level of co-expression of the known genes. We have already successfully applied this method to predict TMEM107 as part of the ciliary transition zone [73] and now extend the approach to the complete cilium. An integrated cilium co-expression data set was constructed by applying the weighted co-expression method WeGet [74] to ciliary genes in 465 human expression data sets available in the NCBI Gene Expression Omnibus [75]. For individual genes, correlations of their expression profiles were determined with expression profiles of the set of ciliary components. The contribution of each data set to a final co-expression score per gene was weighed by how consistently the set of cilia components were expressed together, i.e. how well the data set in question is able to detect ciliary components [74]. To avoid circularity with the training of the Bayesian classifier the expression screen was performed using a gene set of ciliary components from GO (GO:Cilium) and removed any overlap from the positive set used to evaluate this data set for the Bayesian classifier. The data set from Ross *et al*.[24], which has been included in the Bayesian classifier, has been excluded from the microarray data sets used in the expression screen.

### Ciliary co-evolution data set

Given the large number of independent losses of the cilium in eukaryotic evolution (we counted eight independent loss events throughout the eukaryotic kingdom) [76–78], presence/absence profiles have a high value for predicting new cilium genes [14,73]. We constructed a comprehensive co-evolution data set from a comparative genomics analysis of presence-absence correlation patterns over a representative data set of eukaryotic species. We correlated the occurrence of orthologs of 22,000 human genes in 52 eukaryotic genomes to that of cilia or flagella using differential Dollo parsimony (DDP)[79]. A perfectly matched profile pair would obtain a DDP of 0 (all events match, no differences), while mismatching profile pairs would receive a DDP equal to the number of evolutionary events that did not occur at the same time in evolution (e.g. the gene was lost in a lineage still maintaining a cilium, or the gene was maintained in a lineage in which the cilium has been lost). Thus we obtained an objective measure for each human gene that describes how well its evolutionary trajectory (i.e. point of origin and independent loss events) matches that of the ciliary system. Due to the topology of the species tree we observed a complicated distribution of genes from the positive and negative sets (S11A Fig): for low DDP scores (0–6) we did not observe a single negative gene, which would result in unrealistic log odd scores (i.e. infinity). To avoid these unrealistic log odds, we decided to combine the DDP scores into two categories, namely genes with a DDP ≤ 9 and genes with a DDP ≥ 10. Ciliary genes are generally overrepresented among genes with a score between 0 and 9 (S11B Fig).

### Transcription factor binding sites data set

The RFX and FOXJ1 transcription factors play an important role in the regulation of ciliogenesis [80,81]. X-box (RFX) or a FOXJ1 transcription factor binding site (TFBS) have been used to predict novel ciliary genes in *Caenorhabditis elegans* (nematode) and *Drosophila melanogaster* (fruit fly) [82–84]. We processed the publicly available data sets from the 29 mammals project[22] to obtain human genes with a conserved X-box or FOXJ1 TFBS in their promoters, which were defined as 4 kilobase (kb) windows centered (i.e. 2kb upstream and 2kb downstream) at all annotated transcription start sites of the gene. The restriction that the TFBS motifs are conserved among mammalian species infers a higher level of confidence that these motifs are indeed relevant and not spurious hits. The final data set was constructed by defining two categories, namely: “Gene has a X-box and/or FOXJ1 TFBS”, represented as 1, and “Gene does not have a X-box and/or FOXJ1 TFBS”, represented as 0. We found relatively limited overlap with the previous invertebrate X-box TFBS data sets: 13% of the genes with an X-box in *C*. *elegans* (225 out of 1695 genes) [82–84] and 15% in *D*. *melanogaster* (71 out of 470)[84] have a conserved X-box in human. This low overlap may result from differences in the sensitivity detecting functional X-box sequences. It might also indicate that the X-box motifs are transient in the genome; i.e. often gained and lost, as has also been observed in vertebrate evolution of other TF binding sites [85].

### Published data sets

There are a number of high-throughput cilia data sets available from the CilDB [17] that can complement the data sets mentioned above. We only included data sets from mammalian species to avoid significant issues with orthology (i.e. avoid mapping to paralogous genes) and which had a broad coverage of the entire genome/proteome (S1 Fig). We excluded data sets that focused specifically on the centriole/basal body, since this structure is also affected by the cell cycle [86,87] and therefore could potentially skew the Bayesian classifier towards this process. We avoided redundancy in the data sets by selecting only one data set per experiment type (e.g. proteomics, expression). We only considered proteomics datasets specifically generated for the cilium as opposed to whole cell proteomics data sets to minimize false positives. The data from Ross *et al*.[24] (expression) and Liu *et al*. [23] (proteomics) were selected based on these criteria. These data sets were extracted from CilDB [17] and implemented using the predefined confidence categories from CilDB (Low confidence, medium confidence, high confidence). Genes not covered by these data sets were assigned to the “not found” category.

### Training sets

We used the SYSCILIA Gold Standard (SCGS) [7] as our positive training set. We did not use GO annotations as at the time we regarded the SCGS, which has been annotated by experts in the cilium field, of higher quality. Furthermore, having an independent cilium genes dataset allowed us to prevent circularity in the expression screening analysis (see below). The SCGS, or positive set, contains a total of 302 manually curated human ciliary genes. For details about the negative set and about measures undertaken to avoid overtraining see S1 File. We constructed a negative set by selecting genes annotated in GO to function in processes and cellular compartments we deemed least likely to be (in)directly involved in ciliary processes. We selected genes annotated with at least one of the following GO Cellular Component terms: extracellular, lysosome, endosome, peroxisome, ribosome, and nucleolus. We ensured that the positive and negative training sets do not overlap by removing genes found in both from the negative set. Since the majority of human genes are expected to be non-ciliary the similarity between the score distribution of the negative set and the remaining “other genes” indicates that the negative set overall gives an excellent representation of what we reasonably can expect to be non-ciliary genes. The final negative set contains 1275 genes.

We adapted the positive training set for the PPI data sets as well as the expression screen data set to avoid overtraining. Since many of the baits used in the PPI data are known ciliary components and therefore part of the positive training set, this could lead to a potential overestimation of the predictive value of the data sets. Therefore, we excluded the bait proteins from the positive set for evaluating the Y2H, TAP-MS and SILAC data. The training of the expression screen was performed using a gene set of ciliary components from GO (GO:Cilium) and to avoid overtraining we subtracted the overlap from the positive set used for the Bayesian classifier. In this way we avoided inflation of the predictive values for these data sets.

### Bayesian classifier

The Bayesian classifier approach was based on Van der Lee et al. [10], which itself was an adoption of the methods as used by Pagliarini et al. [11]. The method, including equations, is discussed in S1 File.

### Validation and OSCP1 experiments

#### Candidate selection

The number of genes tested per method, the model organisms and cell-lines were determined by time and resources available to the participating labs. Orthologs in *C*. *elegans* were identified for the candidate list. For 21 orthologs null alleles were obtained from the *Caenorhabditis* Genetics Center (University of Minnesota, USA) and tested. Candidates for the eCFP localization studies in hTERT-RPE1 cells were randomly selected. The candidates for the immunofluorescence in human lung epithelial and murine retina were selected based on the availability of a suitable antibody in the collaborating labs. Candidates tested in zebrafish were randomly selected based on the presence of unambiguous 1–1 orthologs. An equal spread of the selected candidates throughout the ranked list was taken into account as much as possible as is shown in Fig 4H.

#### Localization studies in hTERT-RPE1 cells

Expression constructs were created with Gateway Technology (Life Technologies) according to the manufacturer’s instructions. These constructs encoded eCFP fusion proteins of OSCP1 (transcript variant 1; NM_145047.4), CFAP61 (C20orf26, NM_015585), CFAP20 (C16orf80, NM_013242.2), CYB5D1 (NM_144607), CCDC147 (M_001008723.1), CFAP161 (C15orf26, NM_173528), IQCA1 (NM_024726.4), IPO5 (NM_002271), HSPA1L (NM_005527), and TEKT1 (NM_053285). The sequences for all entry clones were verified by Sanger sequencing. Human TERT-immortalized retinal pigment epithelium 1 (hTERT- RPE1) cells were cultured as previously described[88]. Cells were seeded on coverslips, grown to 80% confluency, and subsequently serum-starved for 24 hr in medium containing only 0.2% foetal calf serum for inducing cilium growth. The cells were then transfected with eCFP expression construct using Lipofectamine 2000 (Life Technologies) according to the manufacturer’s instructions. Cells were fixed in 4% paraformaldehyde for 20 min, treated with 1% Triton X-100 in PBS for 5 min, and blocked in 2% BSA in PBS for 20 min. Cells were incubated with the primary antibody GT335 (cilium and basal body marker, 1:500) diluted in 2% BSA in PBS, for 1 hr. After washing in PBS, the cells were incubated with the secondary antibody for 45 min. Secondary antibody, goat anti-mouse Alexa 568 (1:500; Life Technologies) was diluted in 2% BSA in PBS. Cells were washed with PBS and briefly with milliQ before being mounted in Vectashield containing DAPI (Vector Laboratories). The cellular localization of eCFP-fused proteins was analyzed with a Zeiss Axio Imager Z1 fluorescence microscope equipped with a 63x objective lens. Optical sections were generated through structured illumination by the insertion of an ApoTome slider into the illumination path and subsequent processing with AxioVision (Zeiss) and Photoshop CS6 (Adobe Systems) software.

#### Immunofluorescence microscopy of cells

IMCD3 and ATDC5 cells were growth to 80% confluence in DMEM-Glutamax medium with 10% Foetal Bovine Serum. Then cells were serum-starved for 24 hours and fixed in cold methanol for 5 minutes, PBS washed and blocked with 1% Bovine Serum Albumin for 1 hour before incubating with primary antibodies overnight at room temperature. Antibodies and concentrations were anti-acetylated tubulin (Sigma 6-11B-1, T7451) 1/200, anti-gamma-tubulin (Sigma GTU-88, T6557) 1/200, anti-OSCP1 Proteintech 12598-1-AP 1/100, anti-OSCP1 ATLAS HPA028436 1/100 and anti-OSCP1 Biorbyt 185681 1/100. Human respiratory cells were analyzed by immunofluorescence microscopy as previously described [89]. The following rabbit polyclonal antibodies were purchased from Atlas antibodies: anti-CCDC113 (HPA040869), anti-RIBC2 (HPA003210), anti-ARMC3 (HPA037824), anti-C6orf165 (HPA044891) and anti-OSCP1 (HPA028436).

#### Mouse handling and experiments

C57Bl/6J wild-type mice were kept on a 12 h light-dark cycle with unlimited access to food and water. All procedures were in accordance with the guidelines set by the ARVO statement for the use of animals in Ophthalmic and Vision Research and the local laws on animal protection. The following antibodies were used for immunofluorescence/immuno-EM analysis of murine retina sections: rabbit anti-CCDC113 (1:500/1:500), anti-RIBC2 (1:250/1:250), anti-EFHC1 (1:500/-), anti-WDR69 (1:250/-), C6orf165 (1:50/1:200), anti-ARMC3 (1:250/1:500), anti-OSCP1 (for IF 1:100, biorbyt, Cambridge, UK; 1:100, proteintech, Manchester, UK; for EM: 1:100, Atlas, Stockholm, Sweden), mouse anti-centrin-3 (1:100; [90]), rabbit anti-rootletin (1:100, [91]). Sections were counterstained with DAPI (1 mg/ml) Sigma-Aldrich, Munich, Germany), and, where applicable, with FITC-labeled peanut agglutinin (PNA, 1:400, Sigma-Aldrich, Munich, Germany). Secondary antibodies conjugated to Alexa 488, Alexa 555, and Alexa 568 (1:400) were purchased from Invitrogen (Karlsruhe, Germany) and CF-640 (1:400) from Biotrend Chemikalien GmbH (Cologne, Germany). For pre-embedding electron microscopy, we used biotinylated secondary antibodies (1:150; Vector Laboratories, Burlingame, CA, USA). For immunofluorescence microscopy, the eyes of adult C57Bl/6J mice were cryofixed sectioned, and immunostained as described previously [92]. We double-stained the cryosections for CCDC113, RIBC2, EFHC1, WDR69, C6orf165, ARMC3, OSCP1, and centrin-3 as a molecular marker for the connecting cilium, the basal body, and the adjacent centriole of photoreceptor cells [93] at 4°C overnight. Sections stained for C6orf165 were counterstained with FITC-labeled cone photoreceptor marker peanut agglutinin (PNA[94–96]). After one-hour incubation at room temperature with the according secondary antibodies and the nuclear marker DAPI, sections were mounted in Mowiol 4.88 (Hoechst, Frankfurt, Germany). Images were obtained and deconvoluted with a Leica LEITZ DM6000B microscope (Leica, Wetzlar, Germany) and processed with Adobe Photoshop CS with respect to contrast and color correction as well as bicubic pixel interpolation. We applied a pre-embedding labeling protocol as previously introduced for immunoelectron microscopy of mouse photoreceptor cells [97–99]. Ultrathin sections were analyzed with a transmission electron microscope (TEM) (Tecnai 12 BioTwin; FEI, Eindhoven, The Netherlands). Images were obtained with a charge-coupled device camera (SIS MegaView3, Olympus, Shinjuka, Japan) and processed with Adobe Photoshop CS (brightness and contrast).

#### Zebrafish handling and experiments

Wild-type (AB × Tup LF) zebrafish were maintained and staged as described previously in [100]. Antisense MO oligonucleotides (Gene Tools) were designed against the start codons and against splice sites, as described in S6 Table. MOs were injected (4–6 ng) into embryos at the 1- to 2-cell stage and reared at 28.5°C until the desired stage. For cilia immunostaining, 6 somite-stage or 24 hpf (hours post fertilization) embryos were dechorionated and fixed in 4% PFA overnight (O/N) at 4°C, dehydrated through 25%, 50% and 75%, methanol/PBT (1% Triton X-100 in PBS) washes and stored in 100% methanol −20°C. The embryos were rehydrated again through 75%, 50% and 25% methanol/PBT washes. Embryos of 24 hpf were permeabilized with Proteinase K (10ug/ml in PBT) for 10 minutes at 37°C, and subsequently refixated in 4% PFA. Prior to immunostaining, embryos were incubated in block buffer (5% goat serum in PBT) blocked with 5% goat serum (in PBT) for 1 h and subsequently incubated O/N at 4oC with mouse monoclonal anti-γ-tubulin (1:200, GTU-88, Sigma) and anti-acetylated tubulin (1:800, 6-11B-1, Sigma) diluted in blocking buffer. Secondary antibodies used were Alexa Fluor goat anti-mouse IgG1 488, Alexa Fluor donkey anti-mouse IgG2b 568, and Alexa Fluor goat anti-mouse IgG2b 594 (Molecular Probes). Nuclei were stained with Hoechst and embryos were mounted in Citofluor. Z-stack images were captured using a Zeiss 710 Confocal Microscope. For rescue experiments, the aforementioned human OSCP1 cDNA was cloned into pCS2+ using gateway technology. OSCP1 plasmids were linearized using NotI and mRNA was synthesized using Ambion mMessage mMachine kit for the sense strand. 100 pg of mRNA was injected into the cell of one cell–stage embryos. These embryos were subsequently injected with 4 ng oscp1 MO at the two-cell stage, and embryos were allowed to develop at 28.5°C.

#### *C*. *elegans* handling and experiments

*C*. *elegans* were maintained and cultured at 20°C using standard techniques. Mutant strains were obtained from the *Caenorhabditis* Genetics Center (University of Minnesota, USA); the alleles used are shown in S2 Table. Assays for dye uptake (DiI), roaming and osmotic avoidance were performed as previously described[27]. Briefly, for the dye-filling assay, worms were placed into a DiI solution (diluted 1:200 with M9 buffer) for 1 hour, allowed to recover on NGM plates, and then imaged (40x objective, Texas Red filter set) on a compound epi-fluorescence microscope (Leica DM5000B), fitted with an Andor EMCCD camera. For the osmotic avoidance assay, young adult worms were placed within a ring-shaped barrier of 8M glycerol and scored during 10 minutes for worms that crossed the barrier. For the roaming assay, single young adult worms were placed for 16 hours onto seeded plates and track coverage assessed using a grid reference. For transmission electron microscopy, *oscp-1(gk699)* worms were first outcrossed 2 times with wild type worms (to remove unlinked mutations), using primers that flank the *gk699* deletion. Day 1 adults were fixed, sectioned and imaged as described previously[27]. Translational reporters were introduced into *oscp-1(gk699)* by standard mating methods. The translational construct for *oscp-1* (R10F2.5 and R10F2.4) was generated by fusing the genomic region, including 853 bp of the native promoter, to GFP with the *unc-54* 3’ UTR. Standard mating procedures were used to introduce OSCP-1::GFP into the *mks-5(tm3100)* mutant background.

## Supporting information

S1 Fig Predictive values (in Bayesian log odds) versus human genome coverage for each mammalian data set available in CilDBv2. The choice for the Liu et al. and Ross et al. data sets is based on several aspects we needed to consider. First is the overall quality of the dataset, second is the coverage, and third is the specific technique used to obtain the data. The data set with the highest predictive value is the McClintock data set, but it has a relatively low coverage. The Liu set is about average in predictive value but has a coverage of over 11%. The data sets that can be included need to be dissimilar in technique and experimental design to meet the independence assumption of the naive Bayesian integration method. Hence, we limited ourselves to two data sets (marked in red) of dissimilar experimental design and technique and chose accordingly based on a balance of quality and coverage.
(PDF)

S2 Fig 10-fold cross validation of the Bayesian classifier. The receiver-operator characteristics curve based on the 10-fold cross validation (red) and has an area under the curve of 0.86 and overlaps with the curve of the classifier trained with the full set (black). The similarity of the distributions of the negative and positive sets for each fold validation indicates that the results are very robust.
(PDF)

S3 Fig Images showing dye uptake in *C*. *elegans* null mutant strains for DMD, MAGI2 and OSCP1. Dye uptake in wild type is provided for comparison.
(PDF)

S4 Fig Localization of nine eCFP fusion constructs of candidate genes in hTERT-RPE1 cells. Per eCFP fusion protein two representative photos are taken, containing at least one ciliated cell from the same slide. Cells transfected for c15orf22::eCFP and c16orf80 ciliated only when expression of the eCFP fusion protein was low. Exposure times used to image the eCFP protein are shown above each panel. For all constructs, except IQCA, ciliary and/or basal body localization could be observed with “optical sectioning using structured illumination” under normal exposure times (100ms-3000ms). Images with digitally increased gamma are shown for IQCA1::eCFP, demonstrating absence of eCFP fusion protein in and around the cilium. Poly glutamylated tubulin (red) is used to color the ciliary axoneme.
(PDF)

S5 Fig Localization of OSCP-1::GFP fusion in *C*. *elegans* amphid and phasmid cilia. a) OSCP-1 localization is not altered in *mks-5(tm3100)* worms with disrupted ciliary transition zones. Shown are phasmid cilia of worms expressing GFP-tagged OSCP-1 and XBX-1::tdTomato (ciliary marker). Scale bar; 5 μm. b) The ciliary transition zone is intact in *oscp-1(gk699)* mutant worms. Shown are images of phasmid cilia of worms expressing markers for transition zone proteins (MKS-5, NPHP-4 and MKS-2), the periciliary membrane (TRAM-1; normally excluded from the ciliary membrane), and the ciliary axoneme (DYF-11 and CHE-11). NPHP-4, MKS-2 and MKS-5 localizations are unaffected in *oscp-1* worms, indicating that the composition of the transition zone is not dramatically affected. TRAM-1 remains excluded from the ciliary membrane of *oscp-1* mutant indicating the membrane diffusion barrier at the transition zone membrane is intact. The ciliary axoneme markers (see also XBX-1 marker in the top panels) show that phasmid cilia are assembled and of grossly normal length in *oscp-1* mutant worms. Scale bar; 5 μm.
(PDF)

S6 Fig Localization of OSCP1 in ciliated murine ATDC5 and IMCD3 cells using three different antibodies. Scale bar; 5 μm.
(PDF)

S7 Fig *oscp1* zebrafish morpholino phenotypes. Zebrafish strong oscp1 mo phenotype: short body, small eye, obvious pronephric cyst development, and obvious heart edemas. Zebrafish weak oscp1 mo phenotype: short body, small eye, small heads, but no obvious pronephric cyst or heart edemas.
(TIF)

S8 Fig Venn diagram showing the overlaps of the individual components of the CiliaCarta resources. GO, the SYSCILIA Gold Standard (SCGS), and the top predictions of our Bayesian integration. Surface size of each circle and overlap corresponds to the size of the set enclosed.
(PDF)

S9 Fig Ultrastructure of amphid channel cilia in *oscp-1* mutant. Shown are transmission electron microscopy (TEM) serial cross-section images of wild-type and *oscp-1(gk699)* amphid channel cilia. Like wild type controls (N2 worms), the amphid channels of *oscp-1* mutants contain a full complement of 10 ciliary axonemes, each demonstrating intact distal segment (DS; singlet A-tubules), middle segment (MS; doublet A/B tubules), transition zone (TZ; with Y-links), and periciliary membrane (PCMC; swelling at distal dendrite ending immediately proximal to the ciliary axoneme) compartments. Also, the integrity of the ciliary membranes and microtubules were normal in *oscp-1* worms. Schematics show the amphid channel in cross section and longitudinal orientations (only 3 axonemes shown for simplicity in longitudinal cartoon). Numbers above images indicate the position of the section relative to the most anterior section (at ‘0’); section positions also indicated in schematic by arrows. Scale bars; 200 nm.
(PDF)

S10 Fig Pairwise correlations of CiliaCarta datasets. a) Pairwise correlations between data sets according to the positive training set. b) Pairwise correlations between data sets according to the negative set.
(PDF)

S11 Fig Cilia co-occurrence frequencies of positive and negative set per DDP score. a) Frequency of training sets per DDP score. For low DDP scores, no negatives were counted, which would result in unrealistic Bayesian log odds for these categories. b) Cumulative frequencies. For x ≤ 9 in figure b the positive set is significantly overrepresented. We therefore used DDP ≤ 9 as a threshold in the Bayesian classifier to define the two sub-categories.
(PDF)

S1 Table CiliaCarta Bayesian scores, gene rank and data table. The table lists the genome wide CiliaCarta Scores and results from the individual data sets integrated. For each human gene the rank, CiliaCarta Score, false discovery rate and data from each data set is listed including whether a gene belongs to the positive (P) or negative set (N).
(XLSX)

S2 Table Table listing all 21 candidate genes tested in *C*. *elegans* for structural cilia defects by the dye-filling essay (Dye uptake), and behavioral defects by roaming (Roaming) and osmotic avoidance (Osm) essays. The N2 strain (wild type) is included as a negative control, while PT709 *nphp-4(tm925)*, *him-5(e1490)*, FX3100 *mks-5(tm3100)*, and PR813 *osm-5(p813)* were included as positive controls. The mutants are scored as follows: “++”normal, “+” mild effect, “-” severe effect. Specifically for roaming: “++” normal, “-”defective (p<0.05 unpaired t-test vs. WT). For dye uptake: “++” normal, “+/++” slightly reduced uptake, “+” mild reduces dye uptake, “-”defective dye uptake. For osmotic avoidance: “++” normal. For NBEA (F10F2.1 *sel-2*) two mutants were tested but only one (Million Mutation Project strain) exhibited the roaming defect.
(XLSX)

S3 Table The CiliaCarta. Contains the combined lists of the SYSCILIA Gold Standard, Gene Ontology and the validated candidate list.
(XLSX)

S4 Table The protein-protein interactions obtained by SILAC. (XLSX)

S5 Table The protein-protein interactions obtained by Y2H cDNA library screens. (XLSX)

S6 Table Zebrafish morpholino oligonucleotides used in this study. (XLSX)

S7 Table Bayesian log odds per data set sub category. (XLSX)

S1 File Information on the Bayesian classifier. (DOCX)

## References

[1] Yuan S, Sun Z. Expanding horizons: ciliary proteins reach beyond cilia. Annu Rev Genet. Annual Reviews; 2013;47: 353–76. 10.1146/annurev-genet-111212-133243 24016188 PMC5703194

[2] Reiter JF, Leroux MR. Genes and molecular pathways underpinning ciliopathies. Nat Rev Mol Cell Biol. Nature Publishing Group; 2017;18: 533–547. 10.1038/nrm.2017.60 28698599 PMC5851292

[3] Waters AM, Beales PL. Ciliopathies: an expanding disease spectrum. Pediatr Nephrol. Springer Berlin / Heidelberg; 2011; 1–18–18. 10.1007/s00467-010-1731-7 21210154 PMC3098370

[4] Tyler KM, Fridberg A, Toriello KM, Olson CL, Cieslak JA, Hazlett TL, et al Flagellar membrane localization via association with lipid rafts. J Cell Sci. 2009;122: 859–66. 10.1242/jcs.037721 19240119 PMC2714428

[5] Takao D, Verhey KJ. Gated entry into the ciliary compartment. Cell Mol Life Sci. 2015; 10.1007/s00018-015-2058-0 26472341 PMC4959937

[6] Ashburner M, Ball CA, Blake JA, Botstein D, Butler H, Cherry JM, et al Gene ontology: tool for the unification of biology. The Gene Ontology Consortium. Nat Genet. Nature America Inc.; 2000;25: 25–9. 10.1038/75556 10802651 PMC3037419

[7] van Dam TJ, Wheway G, Slaats GG, Huynen MA, Giles RH. The SYSCILIA gold standard (SCGSv1) of known ciliary components and its applications within a systems biology consortium. Cilia. BioMed Central Ltd; 2013;2: 7 10.1186/2046-2530-2-7 23725226 PMC3674929

[8] Josic D, Clifton JG. Mammalian plasma membrane proteomics. Proteomics. 2007;7: 3010–29. 10.1002/pmic.200700139 17654460

[9] Tabach Y, Billi AC, Hayes GD, Newman MA, Zuk O, Gabel H, et al Identification of small RNA pathway genes using patterns of phylogenetic conservation and divergence. Nature. 2013;493: 694–8. 10.1038/nature11779 23364702 PMC3762460

[10] van der Lee R, Feng Q, Langereis MA, ter Horst R, Szklarczyk R, Netea MG, et al Integrative Genomics-Based Discovery of Novel Regulators of the Innate Antiviral Response. Peters B, editor. PLOS Comput Biol. Public Library of Science; 2015;11: e1004553 10.1371/journal.pcbi.1004553 26485378 PMC4618338

[11] Pagliarini DJ, Calvo SE, Chang B, Sheth SA, Vafai SB, Ong S-E, et al A mitochondrial protein compendium elucidates complex I disease biology. Cell. 2008;134: 112–23. 10.1016/j.cell.2008.06.016 18614015 PMC2778844

[12] Calvo SE, Clauser KR, Mootha VK. MitoCarta2.0: an updated inventory of mammalian mitochondrial proteins. Nucleic Acids Res. 2015; gkv1003. 10.1093/nar/gkv1003 26450961 PMC4702768

[13] Avidor-Reiss T, Maer AM, Koundakjian E, Polyanovsky A, Keil T, Subramaniam S, et al Decoding Cilia FunctionDefining Specialized Genes Required for Compartmentalized Cilia Biogenesis. Cell. 2004;117: 527–539. 10.1016/S0092-8674(04)00412-X 15137945

[14] Li JB, Gerdes JM, Haycraft CJ, Fan Y, Teslovich TM, May-Simera H, et al Comparative Genomics Identifies a Flagellar and Basal Body Proteome that Includes the BBS5 Human Disease Gene. Cell. 2004;117: 541–552. 10.1016/S0092-8674(04)00450-7 15137946

[15] Piasecki BP, Burghoorn J, Swoboda P. Regulatory Factor X (RFX)-mediated transcriptional rewiring of ciliary genes in animals. Proc Natl Acad Sci U S A. 2010;107: 12969–74. 10.1073/pnas.0914241107 20615967 PMC2919930

[16] Ivliev AE, ‘t Hoen PAC, van Roon-Mom WMC, Peters DJM, Sergeeva MG. Exploring the Transcriptome of Ciliated Cells Using In Silico Dissection of Human Tissues. Dias-Neto E, editor. PLoS One. Public Library of Science; 2012;7: e35618 10.1371/journal.pone.0035618 22558177 PMC3338421

[17] Arnaiz O, Malinowska A, Klotz C, Sperling L, Dadlez M, Koll F, et al Cildb: a knowledgebase for centrosomes and cilia. Database. 2009;2009: bap022. 10.1093/database/bap022 20428338 PMC2860946

[18] Touw WG, Bayjanov JR, Overmars L, Backus L, Boekhorst J, Wels M, et al Data mining in the Life Sciences with Random Forest: a walk in the park or lost in the jungle? Brief Bioinform. 2013;14: 315–26. 10.1093/bib/bbs034 22786785 PMC3659301

[19] Boldt K, Van Reeuwijk J, Lu Q, Koutroumpas K, Nguyen T-MT-MT, Texier Y, et al An organelle-specific protein landscape identifies novel diseases and molecular mechanisms. Nat Commun. 2016;7: 11491 10.1038/ncomms11491 27173435 PMC4869170

[20] Kobayashi Y, Tsuchiya A, Hayashi T, Kohyama N, Ohbayashi M, Yamamoto T. Isolation and characterization of polyspecific mouse organic solute carrier protein 1 (mOscp1). Drug Metab Dispos. 2007;35: 1239–45. 10.1124/dmd.107.014795 17446263

[21] Nie X, Zhang B, Li X, Xiang J, Xiao B, Ma J, et al Cloning, expression, and mutation analysis of NOR1, a novel human gene down-regulated in HNE1 nasopharyngeal carcinoma cell line. J Cancer Res Clin Oncol. 2003;129: 410–4. 10.1007/s00432-003-0451-9 12819961 PMC12161968

[22] Lindblad-Toh K, Garber M, Zuk O, Lin MF, Parker BJ, Washietl S, et al A high-resolution map of human evolutionary constraint using 29 mammals. Nature. Nature Publishing Group, a division of Macmillan Publishers Limited. All Rights Reserved.; 2011;478: 476–82. 10.1038/nature10530 21993624 PMC3207357

[23] Liu Q, Tan G, Levenkova N, Li T, Pugh EN, Rux JJ, et al The proteome of the mouse photoreceptor sensory cilium complex. Mol Cell Proteomics. 2007;6: 1299–317. 10.1074/mcp.M700054-MCP200 17494944 PMC2128741

[24] Ross AJ, Dailey LA, Brighton LE, Devlin RB. Transcriptional profiling of mucociliary differentiation in human airway epithelial cells. Am J Respir Cell Mol Biol. 2007;37: 169–85. 10.1165/rcmb.2006-0466OC 17413031

[25] Inglis PN, Ou G, Leroux MR, Scholey JM. The sensory cilia of Caenorhabditis elegans. WormBook. 2007; 1–22. 10.1895/wormbook.1.126.2 18050505 PMC10083726

[26] Starich TA, Herman RK, Kari CK, Yeh WH, Schackwitz WS, Schuyler MW, et al Mutations affecting the chemosensory neurons of Caenorhabditis elegans. Genetics. 1995;139: 171–188. 7705621 10.1093/genetics/139.1.171 PMC1206316

[27] Sanders AAWM, Kennedy J, Blacque OE. Image analysis of Caenorhabditis elegans ciliary transition zone structure, ultrastructure, molecular composition, and function. Methods Cell Biol. 2015;127: 323–47. 10.1016/bs.mcb.2015.01.010 25837399

[28] Coon BG, Hernandez V, Madhivanan K, Mukherjee D, Hanna CB, Barinaga-Rementeria Ramirez I, et al The Lowe syndrome protein OCRL1 is involved in primary cilia assembly. Hum Mol Genet. 2012;21: 1835–47. 10.1093/hmg/ddr615 22228094

[29] Hernandez-Hernandez V, Pravincumar P, Diaz-Font A, May-Simera H, Jenkins D, Knight M, et al Bardet-Biedl syndrome proteins control the cilia length through regulation of actin polymerization. Hum Mol Genet. 2013;22: 3858–68. 10.1093/hmg/ddt241 23716571 PMC3766180

[30] Kok FO, Shin M, Ni C-W, Gupta A, Grosse AS, van Impel A, et al Reverse Genetic Screening Reveals Poor Correlation between Morpholino-Induced and Mutant Phenotypes in Zebrafish. Dev Cell. 2015;32: 97–108. 10.1016/j.devcel.2014.11.018 25533206 PMC4487878

[31] McClintock TS, Glasser CE, Bose SC, Bergman DA. Tissue expression patterns identify mouse cilia genes. Physiol Genomics. 2008;32: 198–206. 10.1152/physiolgenomics.00128.2007 17971504

[32] Koschützke L, Bertram J, Hartmann B, Bartsch D, Lotze M, von Bohlen und Halbach O. SrGAP3 knockout mice display enlarged lateral ventricles and specific cilia disturbances of ependymal cells in the third ventricle. Cell Tissue Res. 2015;361: 645–50. 10.1007/s00441-015-2224-6 26104135

[33] Fujiwara M, Sengupta P, McIntire SL. Regulation of body size and behavioral state of C. elegans by sensory perception and the EGL-4 cGMP-dependent protein kinase. Neuron. 2002;36: 1091–102. 12495624 10.1016/s0896-6273(02)01093-0

[34] Masyukova S V, Winkelbauer ME, Williams CL, Pieczynski JN, Yoder BK. Assessing the pathogenic potential of human Nephronophthisis disease-associated NPHP-4 missense mutations in C. elegans. Hum Mol Genet. 2011;20: 2942–54. 10.1093/hmg/ddr198 21546380 PMC3131040

[35] Williams CL, Winkelbauer ME, Schafer JC, Michaud EJ, Yoder BK. Functional redundancy of the B9 proteins and nephrocystins in Caenorhabditis elegans ciliogenesis. Mol Biol Cell. 2008;19: 2154–68. 10.1091/mbc.E07-10-1070 18337471 PMC2366840

[36] Chung M-I, Kwon T, Tu F, Brooks ER, Gupta R, Meyer M, et al Coordinated genomic control of ciliogenesis and cell movement by RFX2. Elife. 2014;3: e01439 10.7554/eLife.01439 24424412 PMC3889689

[37] Pausch H, Venhoranta H, Wurmser C, Hakala K, Iso-Touru T, Sironen A, et al A frameshift mutation in ARMC3 is associated with a tail stump sperm defect in Swedish Red (Bos taurus) cattle. BMC Genet. BioMed Central; 2016;17: 49 10.1186/s12863-016-0356-7 26923438 PMC4770540

[38] Firat-Karalar EN, Sante J, Elliott S, Stearns T. Proteomic analysis of mammalian sperm cells identifies new components of the centrosome. J Cell Sci. 2014;127: 4128–33. 10.1242/jcs.157008 25074808 PMC4179487

[39] Huu NT, Yoshida H, Yamaguchi M. Tumor suppressor gene OSCP1/NOR1 regulates apoptosis, proliferation, differentiation, and ROS generation during eye development of Drosophila melanogaster. FEBS J. 2015;282: 4727–46. 10.1111/febs.13528 26411401

[40] Shan Z, Hou Q, Zhang N, Guo L, Zhang X, Ma Y, et al Overexpression of oxidored-nitro domain containing protein 1 induces growth inhibition and apoptosis in human prostate cancer PC3 cells. Oncol Rep. 2014;32: 1939–46. 10.3892/or.2014.3407 25118646

[41] Izuno H, Kobayashi Y, Sanada Y, Nihei D, Suzuki M, Kohyama N, et al Rat organic solute carrier protein 1 (rOscp1) mediated the transport of organic solutes in Xenopus laevis oocytes: isolation and pharmacological characterization of rOscp1. Life Sci. 2007;81: 1183–92. 10.1016/j.lfs.2007.08.012 17884105

[42] Hiratsuka K, Yin S-A, Ohtomo T, Fujita M, Ohtsuki K, Isaka H, et al Intratesticular localization of the organic solute carrier protein, OSCP1, in spermatogenic cells in mice. Mol Reprod Dev. 2008;75: 1495–504. 10.1002/mrd.20893 18324622

[43] Huu NT, Yoshida H, Umegawachi T, Miyata S, Yamaguchi M. Structural characterization and subcellular localization of Drosophila organic solute carrier partner 1. BMC Biochem. 2014;15: 11 10.1186/1471-2091-15-11 24939707 PMC4074837

[44] Hiratsuka K, Momose A, Takagi N, Sasaki H, Yin S-A, Fujita M, et al Neuronal expression, cytosolic localization, and developmental regulation of the organic solute carrier partner 1 in the mouse brain. Histochem Cell Biol. 2011;135: 229–38. 10.1007/s00418-011-0790-6 21331566

[45] Jensen VL, Li C, Bowie R V, Clarke L, Mohan S, Blacque OE, et al Formation of the transition zone by Mks5/Rpgrip1L establishes a ciliary zone of exclusion (CIZE) that compartmentalises ciliary signalling proteins and controls PIP2 ciliary abundance. EMBO J. 2015;34: 2537–56. 10.15252/embj.201488044 26392567 PMC4609185

[46] Gherman A, Davis EE, Katsanis N. The ciliary proteome database: an integrated community resource for the genetic and functional dissection of cilia. Nat Genet. 2006;38: 961–2. 10.1038/ng0906-961 16940995

[47] Chevrier V, Bruel A-L, Van Dam TJP, Franco B, Lo Scalzo M, Lembo F, et al OFIP/KIAA0753 forms a complex with OFD1 and FOR20 at pericentriolar satellites and centrosomes and is mutated in one individual with oral-facial-digital syndrome. Hum Mol Genet. 2016;25: 497–513. 10.1093/hmg/ddv488 26643951

[48] Slaats GG, Wheway G, Foletto V, Szymanska K, van Balkom BWM, Logister I, et al Screen-based identification and validation of four new ion channels as regulators of renal ciliogenesis. J Cell Sci. 2015;128: 4550–9. 10.1242/jcs.176065 26546361 PMC4696498

[49] Seeley ES, Nachury M V. Constructing and deconstructing roles for the primary cilium in tissue architecture and cancer. Methods Cell Biol. 2009;94: 299–313. 10.1016/S0091-679X(08)94015-2 20362097 PMC2885964

[50] Michaud EJ, Yoder BK. The primary cilium in cell signaling and cancer. Cancer Res. 2006;66: 6463–7. 10.1158/0008-5472.CAN-06-0462 16818613

[51] Basten SG, Giles RH. Functional aspects of primary cilia in signaling, cell cycle and tumorigenesis. Cilia. 2013;2: 6 10.1186/2046-2530-2-6 23628112 PMC3662159

[52] Litchfield K, Levy M, Dudakia D, Proszek P, Shipley C, Basten S, et al Rare disruptive mutations in ciliary function genes contribute to testicular cancer susceptibility. Nat Commun. 2016;7: 13840 10.1038/ncomms13840 27996046 PMC5187424

[53] Yates A, Akanni W, Amode MR, Barrell D, Billis K, Carvalho-Silva D, et al Ensembl 2016. Nucleic Acids Res. 2015;44: D710–6. 10.1093/nar/gkv1157 26687719 PMC4702834

[54] Kent WJ. BLAT—the BLAST-like alignment tool. Genome Res. 2002;12: 656–64. 10.1101/gr.229202 Article published online before March 2002 11932250 PMC187518

[55] Kent WJ, Sugnet CW, Furey TS, Roskin KM, Pringle TH, Zahler AM, et al The human genome browser at UCSC. Genome Res. 2002;12: 996–1006. 10.1101/gr.229102 Article published online before print in May 2002 12045153 PMC186604

[56] Letteboer SJF, Roepman R. Versatile screening for binary protein-protein interactions by yeast two-hybrid mating. Methods Mol Biol. 2008;484: 145–59. 10.1007/978-1-59745-398-1_10 18592178

[57] Boldt K, Mans DA, Won J, van Reeuwijk J, Vogt A, Kinkl N, et al Disruption of intraflagellar protein transport in photoreceptor cilia causes Leber congenital amaurosis in humans and mice. J Clin Invest. 2011;121: 2169–80. 10.1172/JCI45627 21606596 PMC3104757

[58] Gloeckner CJ, Boldt K, Ueffing M. Strep/FLAG tandem affinity purification (SF-TAP) to study protein interactions. Curr Protoc Protein Sci. 2009;Chapter 19: Unit19.20 10.1002/0471140864.ps1920s57 19688738

[59] Boldt K, van Reeuwijk J, Gloeckner CJ, Ueffing M, Roepman R. Tandem affinity purification of ciliopathy-associated protein complexes. Methods Cell Biol. 2009;91: 143–60. 10.1016/S0091-679X(08)91009-8 20409786

[60] Olsen J V, de Godoy LMF, Li G, Macek B, Mortensen P, Pesch R, et al Parts per million mass accuracy on an Orbitrap mass spectrometer via lock mass injection into a C-trap. Mol Cell Proteomics. 2005;4: 2010–21. 10.1074/mcp.T500030-MCP200 16249172

[61] Cox J, Mann M. MaxQuant enables high peptide identification rates, individualized p.p.b.-range mass accuracies and proteome-wide protein quantification. Nat Biotechnol. 2008;26: 1367–72. 10.1038/nbt.1511 19029910

[62] Cevik S, Sanders AAWM, Van Wijk E, Boldt K, Clarke L, van Reeuwijk J, et al Active transport and diffusion barriers restrict Joubert Syndrome-associated ARL13B/ARL-13 to an Inv-like ciliary membrane subdomain. PLoS Genet. 2013;9: e1003977 10.1371/journal.pgen.1003977 24339792 PMC3854969

[63] Dona M, Bachmann-Gagescu R, Texier Y, Toedt G, Hetterschijt L, Tonnaer EL, et al NINL and DZANK1 Co-function in Vesicle Transport and Are Essential for Photoreceptor Development in Zebrafish. PLoS Genet. 2015;11: e1005574 10.1371/journal.pgen.1005574 26485514 PMC4617706

[64] Chaki M, Airik R, Ghosh AK, Giles RH, Chen R, Slaats GG, et al Exome capture reveals ZNF423 and CEP164 mutations, linking renal ciliopathies to DNA damage response signaling. Cell. 2012;150: 533–48. 10.1016/j.cell.2012.06.028 22863007 PMC3433835

[65] Coene KLM, Roepman R, Doherty D, Afroze B, Kroes HY, Letteboer SJF, et al OFD1 Is Mutated in X-Linked Joubert Syndrome and Interacts with LCA5-Encoded Lebercilin. Am J Hum Genet. 2009;85: 465–481. 10.1016/j.ajhg.2009.09.002 19800048 PMC2756557

[66] Roepman R, Bernoud-Hubac N, Schick DE, Maugeri A, Berger W, Ropers HH, et al The retinitis pigmentosa GTPase regulator (RPGR) interacts with novel transport-like proteins in the outer segments of rod photoreceptors. Hum Mol Genet. 2000;9: 2095–105. 10958648 10.1093/hmg/9.14.2095

[67] Roepman R, Letteboer SJF, Arts HH, van Beersum SEC, Lu X, Krieger E, et al Interaction of nephrocystin-4 and RPGRIP1 is disrupted by nephronophthisis or Leber congenital amaurosis-associated mutations. Proc Natl Acad Sci. 2005;102: 18520–18525. 10.1073/pnas.0505774102 16339905 PMC1317916

[68] Otto EA, Hurd TW, Airik R, Chaki M, Zhou W, Stoetzel C, et al Candidate exome capture identifies mutation of SDCCAG8 as the cause of a retinal-renal ciliopathy. Nat Genet. 2010;42: 840–850. 10.1038/ng.662 20835237 PMC2947620

[69] Kersten FF, van Wijk E, Hetterschijt L, Bauβ K, Peters TA, Aslanyan MG, et al The mitotic spindle protein SPAG5/Astrin connects to the Usher protein network postmitotically. Cilia. 2012;1: 2 10.1186/2046-2530-1-2 23351521 PMC3541543

[70] Eblimit A, Nguyen T-MT, Chen Y, Esteve-Rudd J, Zhong H, Letteboer S, et al Spata7 is a retinal ciliopathy gene critical for correct RPGRIP1 localization and protein trafficking in the retina. Hum Mol Genet. 2015;24: 1584–601. 10.1093/hmg/ddu573 25398945 PMC4351378

[71] Huang L, Szymanska K, Jensen VL, Janecke AR, Innes AM, Davis EE, et al TMEM237 is mutated in individuals with a Joubert syndrome related disorder and expands the role of the TMEM family at the ciliary transition zone. Am J Hum Genet. 2011;89: 713–30. 10.1016/j.ajhg.2011.11.005 22152675 PMC3234373

[72] Baughman JM, Nilsson R, Gohil VM, Arlow DH, Gauhar Z, Mootha VK. A computational screen for regulators of oxidative phosphorylation implicates SLIRP in mitochondrial RNA homeostasis. PLoS Genet. 2009;5: e1000590 10.1371/journal.pgen.1000590 19680543 PMC2721412

[73] Lambacher NJ, Bruel A-L, van Dam TJP, Szymańska K, Slaats GG, Kuhns S, et al TMEM107 recruits ciliopathy proteins to subdomains of the ciliary transition zone and causes Joubert syndrome. Nat Cell Biol. Nature Publishing Group; 2016;18: 122–31. 10.1038/ncb3273 26595381 PMC5580800

[74] Szklarczyk R, Megchelenbrink W, Cizek P, Ledent M, Velemans G, Szklarczyk D, et al WeGET: predicting new genes for molecular systems by weighted co-expression. Nucleic Acids Res. Oxford University Press; 2015; gkv1228. 10.1093/nar/gkv1228 26582928 PMC4702868

[75] Edgar R, Domrachev M, Lash AE. Gene Expression Omnibus: NCBI gene expression and hybridization array data repository. Nucleic Acids Res. 2002;30: 207–10. 10.1093/nar/30.1.207 11752295 PMC99122

[76] van Dam TJP, Townsend MJ, Turk M, Schlessinger A, Sali A, Field MC, et al Evolution of modular intraflagellar transport from a coatomer-like progenitor. Proc Natl Acad Sci U S A. 2013;110: 6943–8. 10.1073/pnas.1221011110 23569277 PMC3637775

[77] Barker AR, Renzaglia KS, Fry K, Dawe HR. Bioinformatic analysis of ciliary transition zone proteins reveals insights into the evolution of ciliopathy networks. BMC Genomics. 2014;15: 531 10.1186/1471-2164-15-531 24969356 PMC4092220

[78] Briggs LJ, Davidge JA, Wickstead B, Ginger ML, Gull K. More than one way to build a flagellum: comparative genomics of parasitic protozoa. Curr Biol. 2004;14: R611–2. 10.1016/j.cub.2004.07.041 15296774

[79] Kensche PR, van Noort V, Dutilh BE, Huynen MA. Practical and theoretical advances in predicting the function of a protein by its phylogenetic distribution. J R Soc Interface. 2008;5: 151–70. 10.1098/rsif.2007.1047 17535793 PMC2405902

[80] Swoboda P, Adler HT, Thomas JH. The RFX-type transcription factor DAF-19 regulates sensory neuron cilium formation in C. elegans. Mol Cell. 2000;5: 411–21. 10882127 10.1016/s1097-2765(00)80436-0

[81] You Y, Huang T, Richer EJ, Schmidt J-EH, Zabner J, Borok Z, et al Role of f-box factor foxj1 in differentiation of ciliated airway epithelial cells. Am J Physiol Lung Cell Mol Physiol. 2004;286: L650–7. 10.1152/ajplung.00170.2003 12818891

[82] Efimenko E, Bubb K, Mak HY, Holzman T, Leroux MR, Ruvkun G, et al Analysis of xbx genes in C. elegans. Development. 2005;132: 1923–34. 10.1242/dev.01775 15790967

[83] Chen N, Mah A, Blacque OE, Chu J, Phgora K, Bakhoum MW, et al Identification of ciliary and ciliopathy genes in Caenorhabditis elegans through comparative genomics. Genome Biol. 2006;7: R126 10.1186/gb-2006-7-12-r126 17187676 PMC1794439

[84] Laurençon A, Dubruille R, Efimenko E, Grenier G, Bissett R, Cortier E, et al Identification of novel regulatory factor X (RFX) target genes by comparative genomics in Drosophila species. Genome Biol. 2007;8: R195 10.1186/gb-2007-8-9-r195 17875208 PMC2375033

[85] Schmidt D, Wilson MD, Ballester B, Schwalie PC, Brown GD, Marshall A, et al Five-vertebrate ChIP-seq reveals the evolutionary dynamics of transcription factor binding. Science. American Association for the Advancement of Science; 2010;328: 1036–40. 10.1126/science.1186176 20378774 PMC3008766

[86] Sorokin S. Centrioles and the formation of rudimentary cilia by fibroblasts and smooth muscle cells. J Cell Biol. 1962;15: 363–77. 13978319 10.1083/jcb.15.2.363 PMC2106144

[87] Hoyer-Fender S. Centriole maturation and transformation to basal body. Semin Cell Dev Biol. 2010;21: 142–7. 10.1016/j.semcdb.2009.07.002 19595783

[88] Graser S, Stierhof Y-D, Lavoie SB, Gassner OS, Lamla S, Le Clech M, et al Cep164, a novel centriole appendage protein required for primary cilium formation. J Cell Biol. 2007;179: 321–30. 10.1083/jcb.200707181 17954613 PMC2064767

[89] Hjeij R, Onoufriadis A, Watson CM, Slagle CE, Klena NT, Dougherty GW, et al CCDC151 mutations cause primary ciliary dyskinesia by disruption of the outer dynein arm docking complex formation. Am J Hum Genet. 2014;95: 257–74. 10.1016/j.ajhg.2014.08.005 25192045 PMC4157146

[90] Trojan P, Krauss N, Choe H-W, Giessl A, Pulvermüller A, Wolfrum U. Centrins in retinal photoreceptor cells: regulators in the connecting cilium. Prog Retin Eye Res. 2008;27: 237–59. 10.1016/j.preteyeres.2008.01.003 18329314

[91] Yang J, Liu X, Yue G, Adamian M, Bulgakov O, Li T. Rootletin, a novel coiled-coil protein, is a structural component of the ciliary rootlet. J Cell Biol. 2002;159: 431–40. 10.1083/jcb.200207153 12427867 PMC2173070

[92] Overlack N, Kilic D, Bauss K, Märker T, Kremer H, van Wijk E, et al Direct interaction of the Usher syndrome 1G protein SANS and myomegalin in the retina. Biochim Biophys Acta. 2011;1813: 1883–92. 10.1016/j.bbamcr.2011.05.015 21767579

[93] Trojan P, Krauss N, Choe H-W, Gießl A, Pulvermüller A, Wolfrum U. Centrins in retinal photoreceptor cells: Regulators in the connecting cilium. Prog Retin Eye Res. Pergamon; 2008;27: 237–259. 10.1016/J.PRETEYERES.2008.01.003 18329314

[94] Blanks JC, Johnson L V. Specific binding of peanut lectin to a class of retinal photoreceptor cells. A species comparison. Invest Ophthalmol Vis Sci. 1984;25: 546–57. 6715128

[95] Reiners J, Märker T, Jürgens K, Reidel B, Wolfrum U. Photoreceptor expression of the Usher syndrome type 1 protein protocadherin 15 (USH1F) and its interaction with the scaffold protein harmonin (USH1C). Mol Vis. 2005;11: 347–55. 15928608

[96] Wunderlich KA, Tanimoto N, Grosche A, Zrenner E, Pekny M, Reichenbach A, et al Retinal functional alterations in mice lacking intermediate filament proteins glial fibrillary acidic protein and vimentin. FASEB J. 2015;29: 4815–28. 10.1096/fj.15-272963 26251181

[97] Sedmak T, Wolfrum U. Intraflagellar transport molecules in ciliary and nonciliary cells of the retina. J Cell Biol. 2010;189: 171–186. 10.1083/jcb.200911095 20368623 PMC2854383

[98] Maerker T, van Wijk E, Overlack N, Kersten FFJ, McGee J, Goldmann T, et al A novel Usher protein network at the periciliary reloading point between molecular transport machineries in vertebrate photoreceptor cells. Hum Mol Genet. 2008;17: 71–86. 10.1093/hmg/ddm285 17906286

[99] Sedmak T, Sehn E, Wolfrum U. Immunoelectron microscopy of vesicle transport to the primary cilium of photoreceptor cells. Methods Cell Biol. 2009;94: 259–72. 10.1016/S0091-679X(08)94013-9 20362095

[100] Westerfield M. The Zebrafish Book: A Guide for the Laboratory Use of Zebrafish (Danio Rerio*). Paperback. Institute of Neuro Science; 1994.

