# Supplementary figures and images for: CiliaCarta: An integrated and validated compendium of ciliary genes
Source: PLoS One. 2019 May 16;14(5):e0216705. doi: 10.1371/journal.pone.0216705 (PMC6522010; doi:10.1371/journal.pone.0216705)

# Coverage versus Bayesian log score of datasets (Hs, Mm, Rn) in CiIDBv2

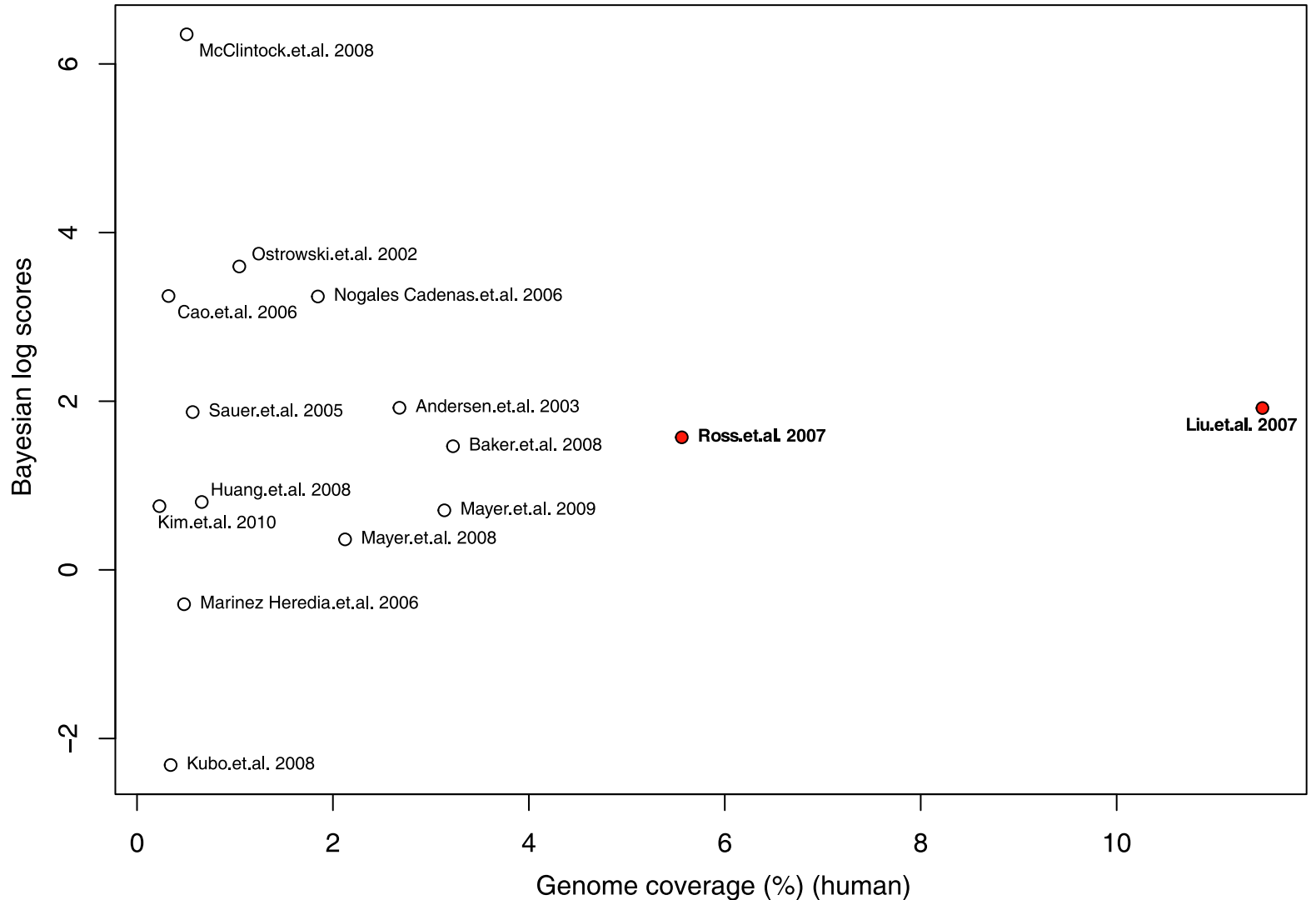

Supplement: S1 Fig — The choice for the Liu et al. and Ross et al. data sets is based on several aspects we needed to consider. First is the overall quality of the dataset, second is the coverage, and third is the specific technique used to obtain the data. The data set with the highest predictive value is the McClintock data set, but it has a relatively low coverage. The Liu set is about average in predictive value but has a coverage of over 11%. The data sets that can be included need to be dissimilar in technique and experimental design to meet the independence assumption of the naive Bayesian integration method. Hence, we limited ourselves to two data sets (marked in red) of dissimilar experimental design and technique and chose accordingly based on a balance of quality and coverage. (PDF) [file pone.0216705.s001.pdf]

A

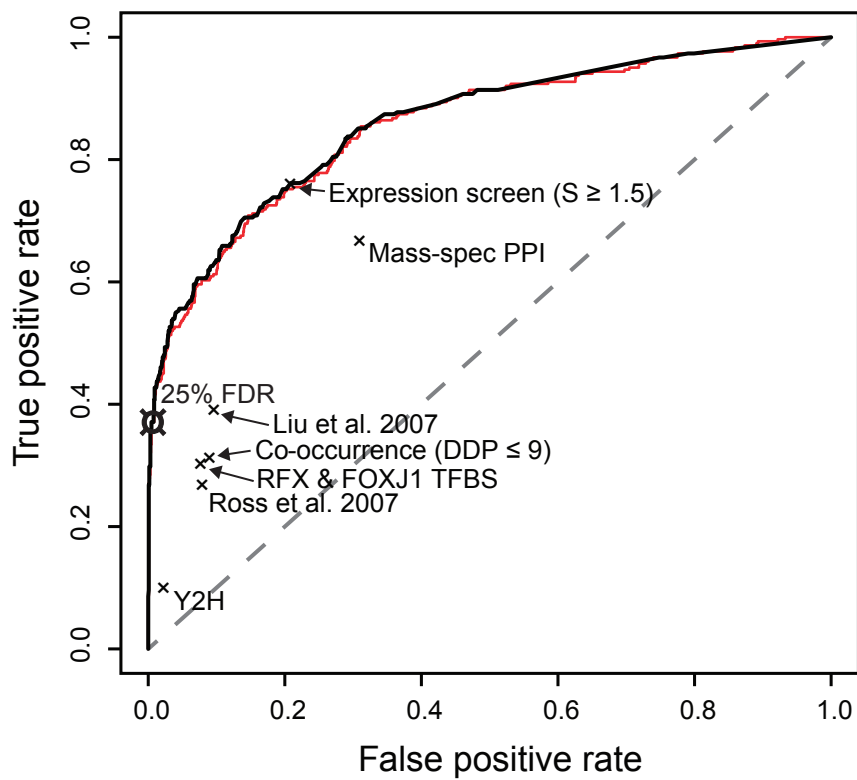

B

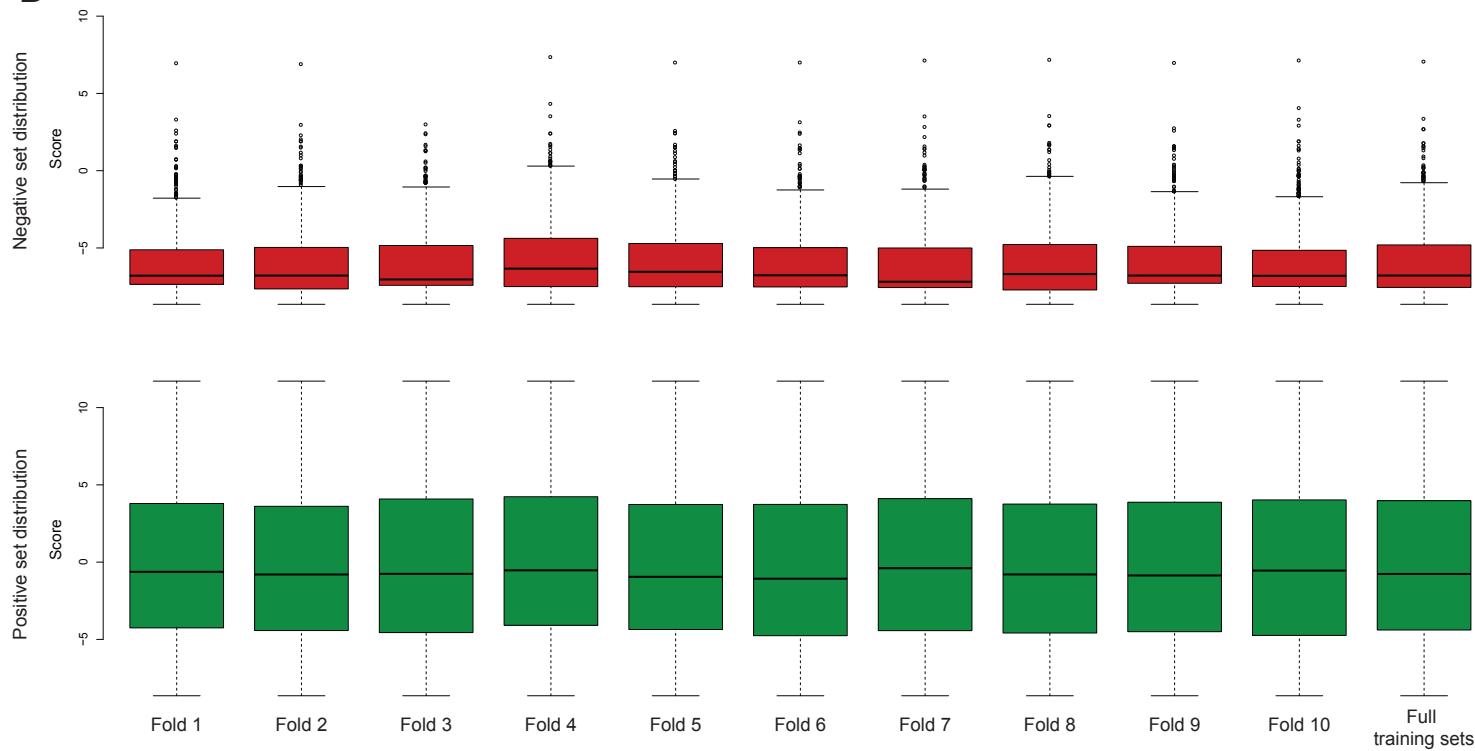

Supplement: S2 Fig — The receiver-operator characteristics curve based on the 10-fold cross validation (red) and has an area under the curve of 0.86 and overlaps with the curve of the classifier trained with the full set (black). The similarity of the distributions of the negative and positive sets for each fold validation indicates that the results are very robust. (PDF) [file pone.0216705.s002.pdf]

*dys-1(cx18)*  
/dystrophin (DMD)

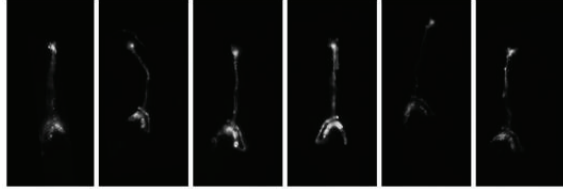

*dys-1(eg33)*  
/dystrophin (DMD)

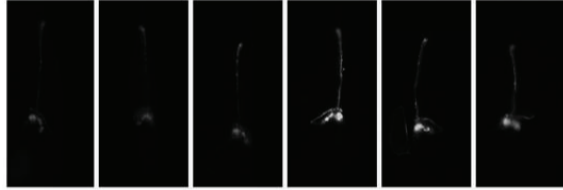

N2 control

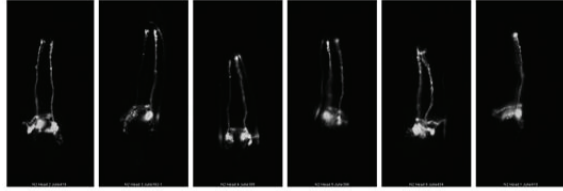

R10F2.5(gk748)  
/OSCP1

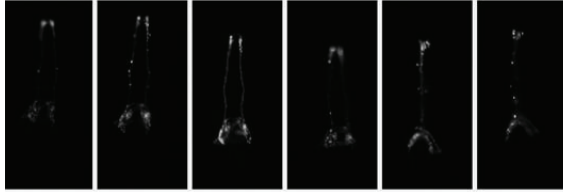

R10F2.5(gk699)  
/OSCP1

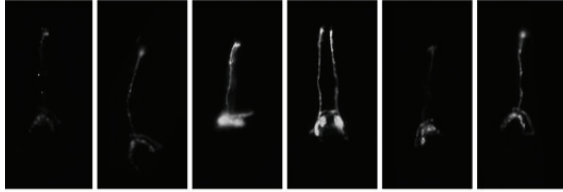

*magi-1(gk657)*  
/MAGI2

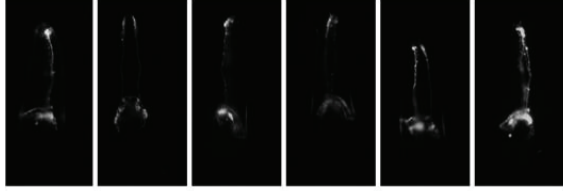

Supplement: S3 Fig — Dye uptake in wild type is provided for comparison. (PDF) [file pone.0216705.s003.pdf]

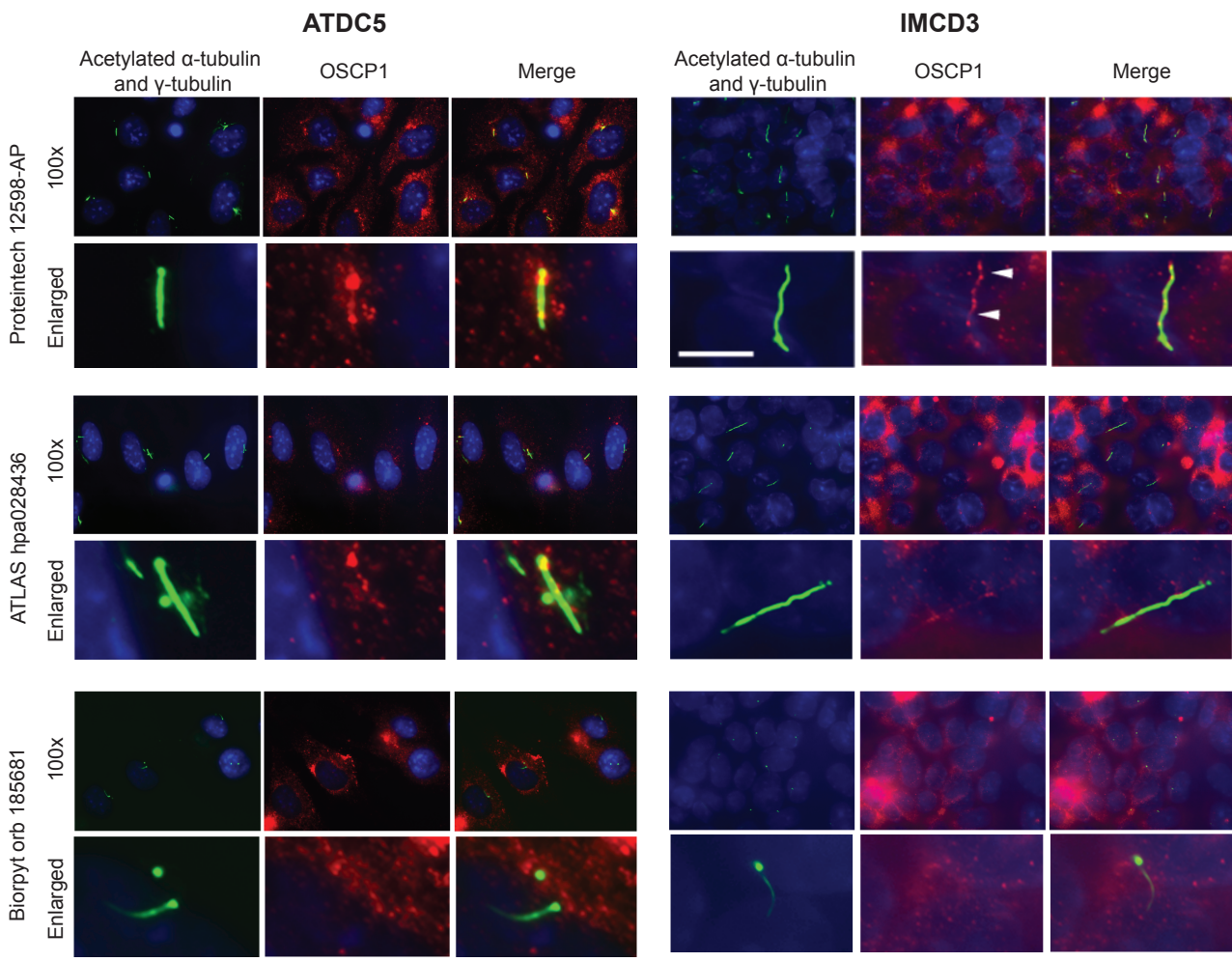

Supplement: S6 Fig — Scale bar; 5 μm. (PDF) [file pone.0216705.s006.pdf]

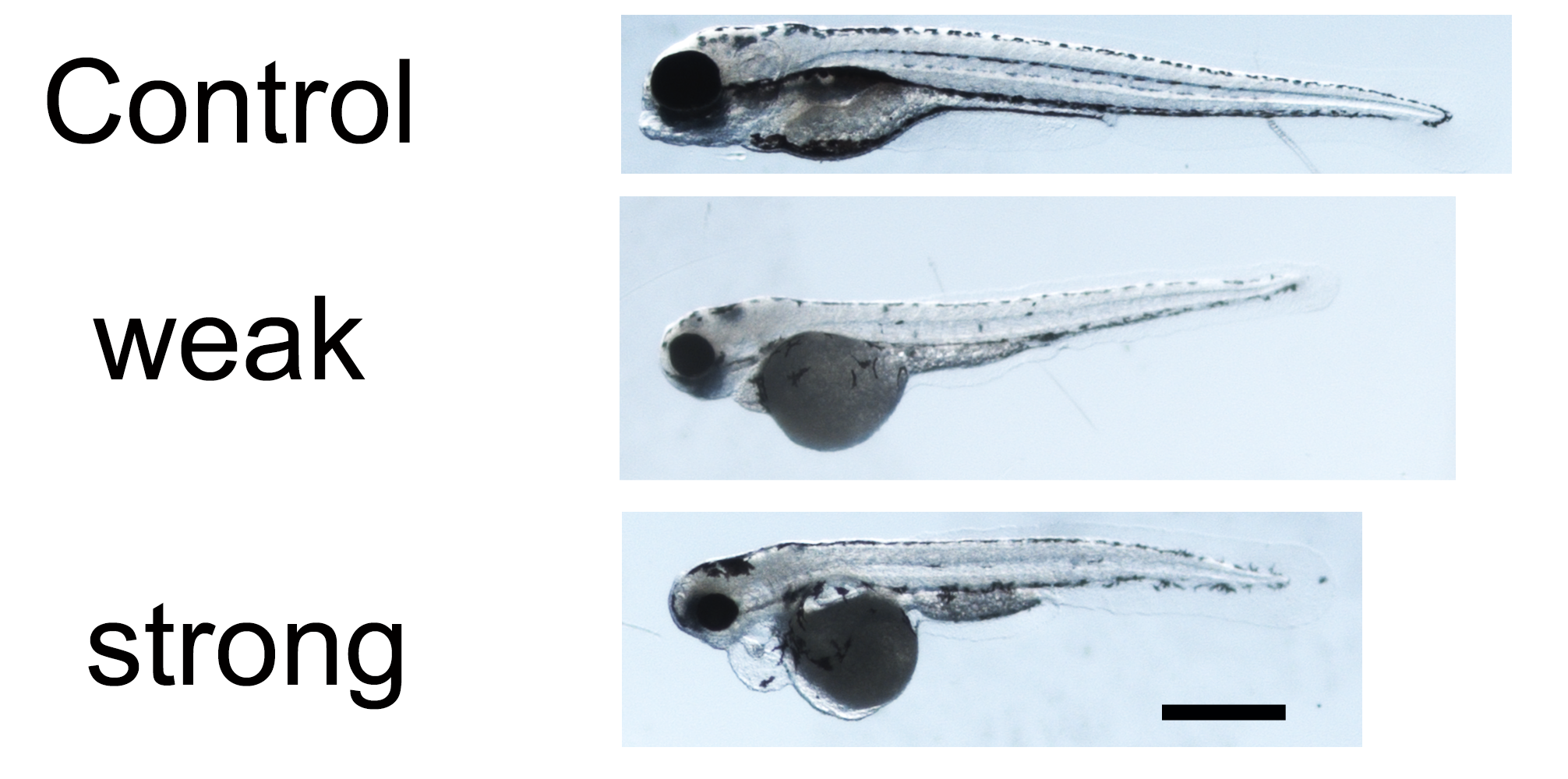

Supplement: S7 Fig — Zebrafish strong oscp1 mo phenotype: short body, small eye, obvious pronephric cyst development, and obvious heart edemas. Zebrafish weak oscp1 mo phenotype: short body, small eye, small heads, but no obvious pronephric cyst or heart edemas. (TIF) [file pone.0216705.s007.tif]

SYSCILIA  
Gold Standard  
(302)

Bayesian predictions  
(285, FDR  $\leq$  25%)

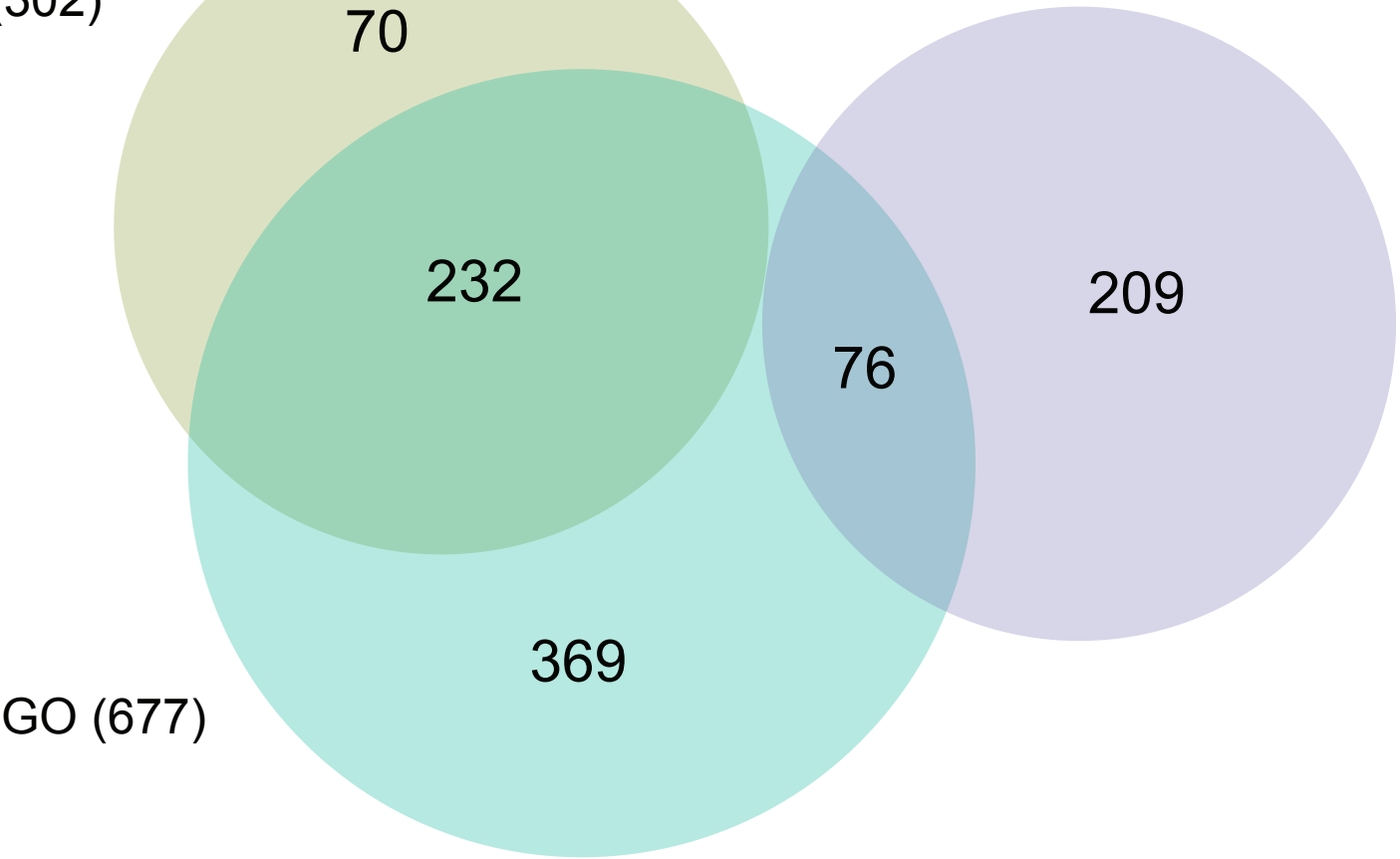

Supplement: S8 Fig — GO, the SYSCILIA Gold Standard (SCGS), and the top predictions of our Bayesian integration. Surface size of each circle and overlap corresponds to the size of the set enclosed. (PDF) [file pone.0216705.s008.pdf]

wild type

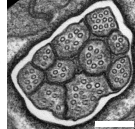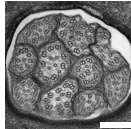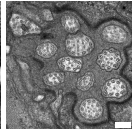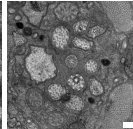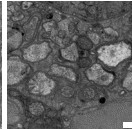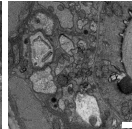

*oscp-1*

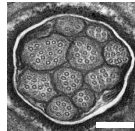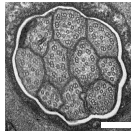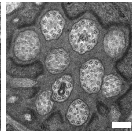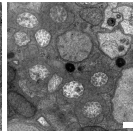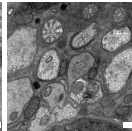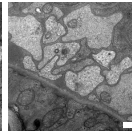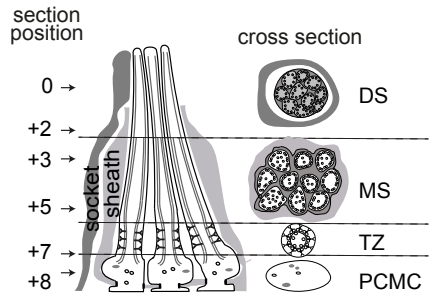

Supplement: S9 Fig — Shown are transmission electron microscopy (TEM) serial cross-section images of wild-type and oscp-1(gk699) amphid channel cilia. Like wild type controls (N2 worms), the amphid channels of oscp-1 mutants contain a full complement of 10 ciliary axonemes, each demonstrating intact distal segment (DS; singlet A-tubules), middle segment (MS; doublet A/B tubules), transition zone (TZ; with Y-links), and periciliary membrane (PCMC; swelling at distal dendrite ending immediately proximal to the ciliary axoneme) compartments. Also, the integrity of the ciliary membranes and microtubules were normal in oscp-1 worms. Schematics show the amphid channel in cross section and longitudinal orientations (only 3 axonemes shown for simplicity in longitudinal cartoon). Numbers above images indicate the position of the section relative to the most anterior section (at ‘0’); section positions also indicated in schematic by arrows. Scale bars; 200 nm. (PDF) [file pone.0216705.s009.pdf]

a

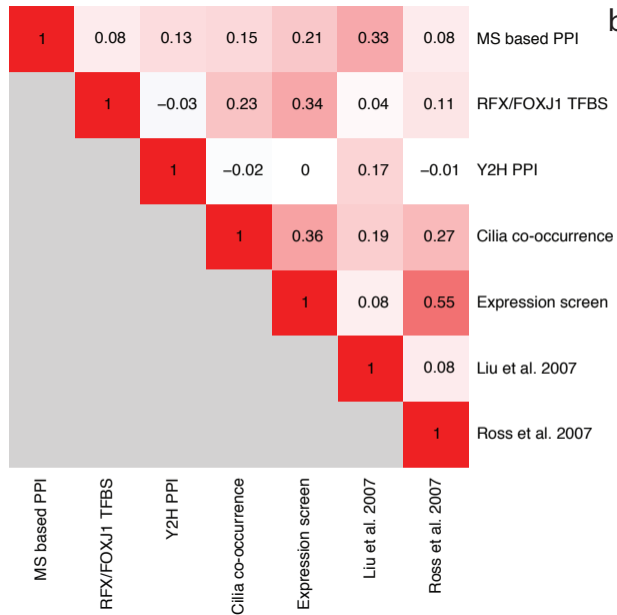

b

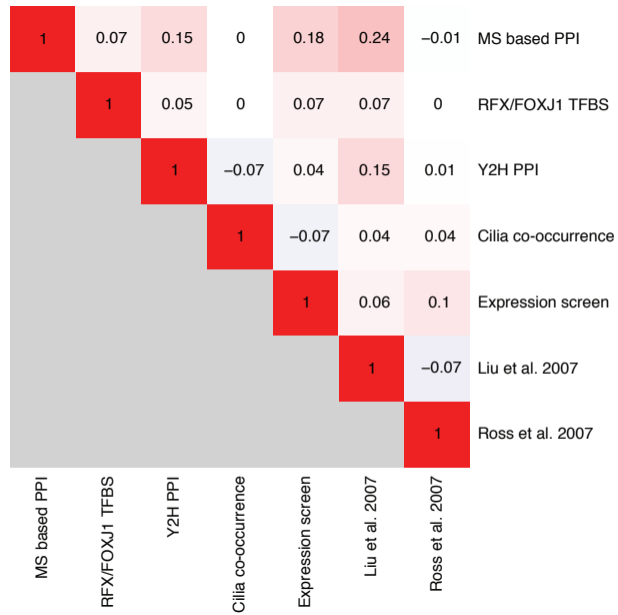

Supplement: S10 Fig — a) Pairwise correlations between data sets according to the positive training set. b) Pairwise correlations between data sets according to the negative set. (PDF) [file pone.0216705.s010.pdf]

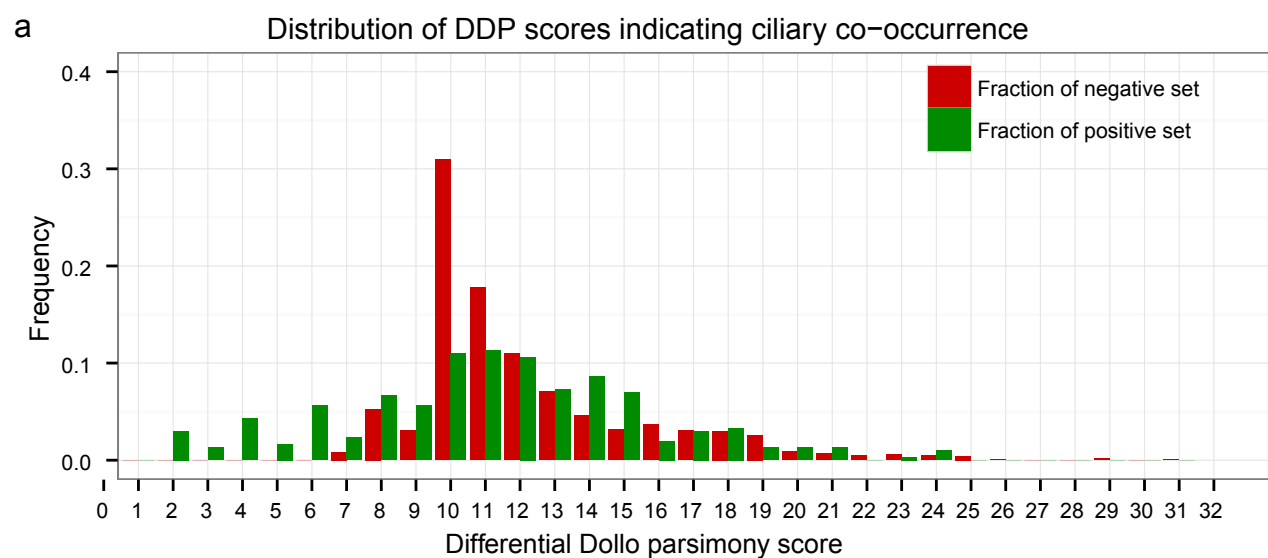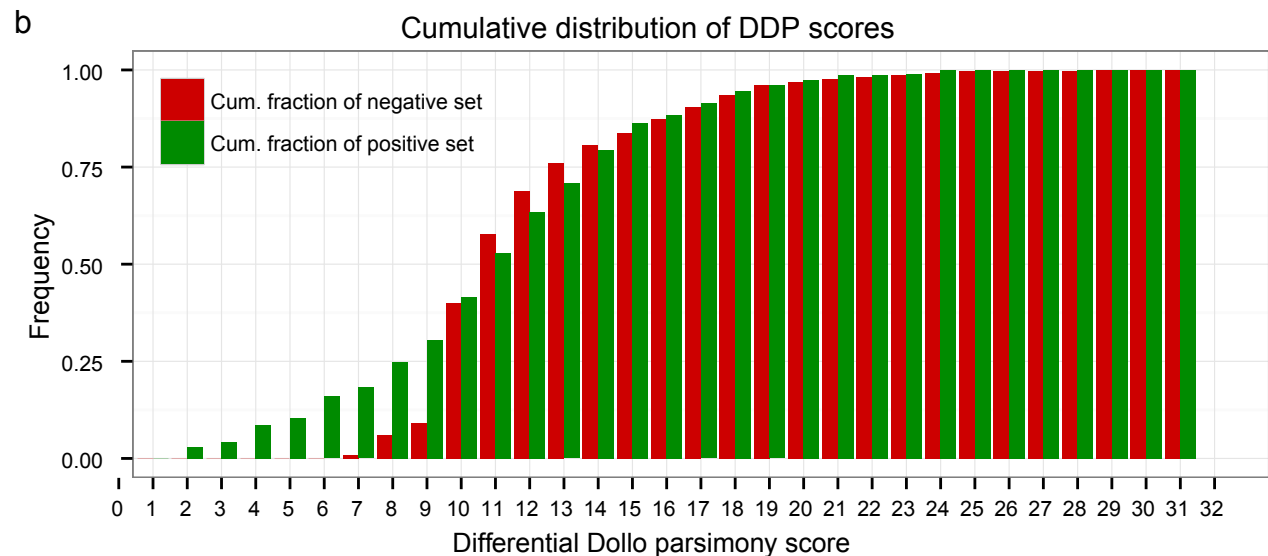

Supplement: S11 Fig — a) Frequency of training sets per DDP score. For low DDP scores, no negatives were counted, which would result in unrealistic Bayesian log odds for these categories. b) Cumulative frequencies. For x ≤ 9 in figure b the positive set is significantly overrepresented. We therefore used DDP ≤ 9 as a threshold in the Bayesian classifier to define the two sub-categories. (PDF) [file pone.0216705.s011.pdf]
