# Supplementary material for: CiliaCarta: An integrated and validated compendium of ciliary genes
Source: PLoS One. 2019 May 16;14(5):e0216705. doi: 10.1371/journal.pone.0216705 (PMC6522010; doi:10.1371/journal.pone.0216705)
Supplement: S4 Fig — Per eCFP fusion protein two representative photos are taken, containing at least one ciliated cell from the same slide. Cells transfected for c15orf22::eCFP and c16orf80 ciliated only when expression of the eCFP fusion protein was low. Exposure times used to image the eCFP protein are shown above each panel. For all constructs, except IQCA, ciliary and/or basal body localization could be observed with “optical sectioning using structured illumination” under normal exposure times (100ms-3000ms). Images with digitally increased gamma are shown for IQCA1::eCFP, demonstrating absence of eCFP fusion protein in and around the cilium. Poly glutamylated tubulin (red) is used to color the ciliary axoneme. (PDF) [file pone.0216705.s004.pdf]

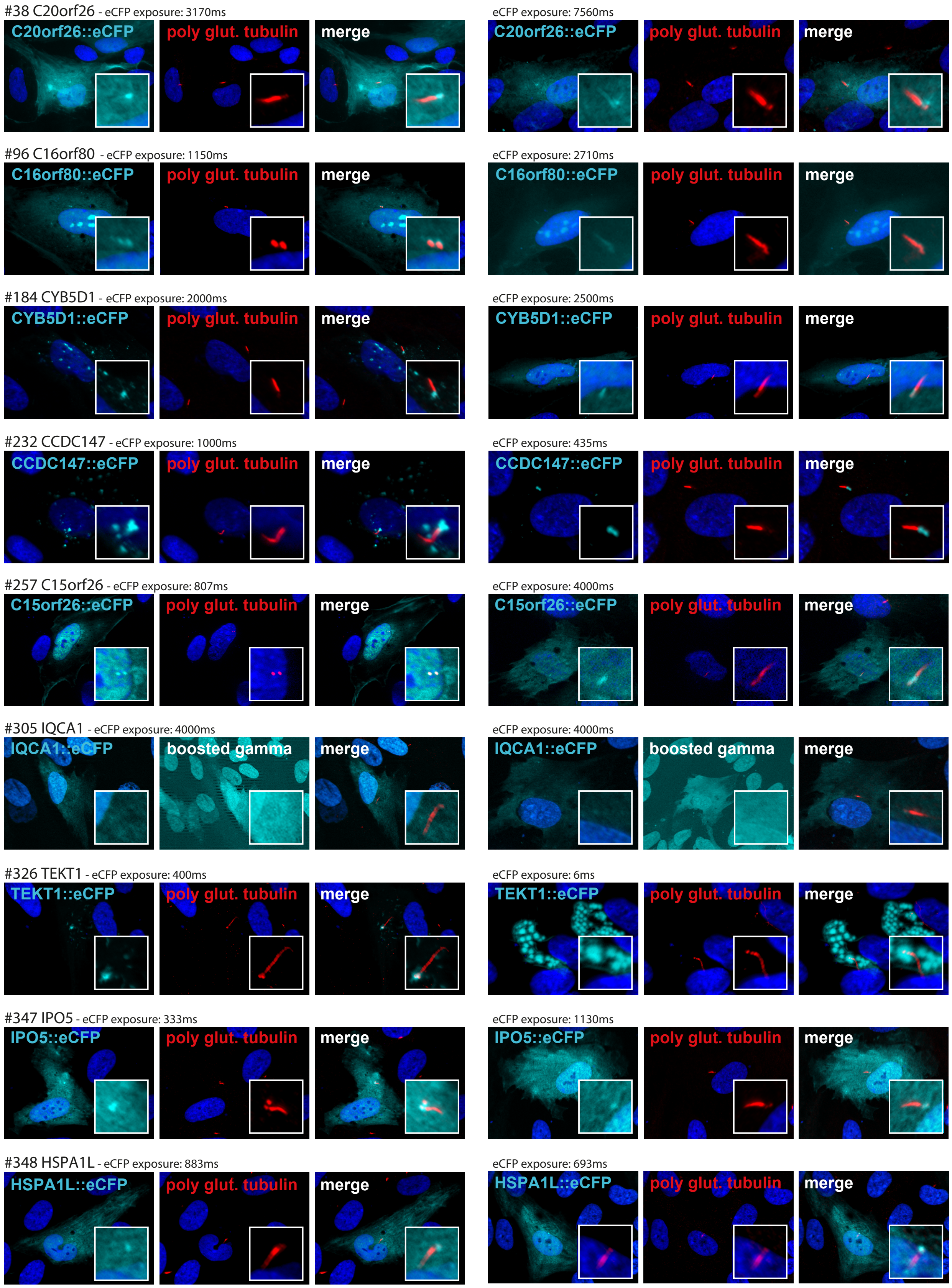

#38 C20orf26 - eCFP exposure: 3170ms
C20orf26::eCFP
poly glut. tubulin
merge
eCFP exposure: 7560ms
C20orf26::eCFP
poly glut. tubulin
merge
#96 C16orf80 - eCFP exposure: 1150ms
C16orf80::eCFP
poly glut. tubulin
merge
eCFP exposure: 2710ms
C16orf80::eCFP
poly glut. tubulin
merge
#184 CYB5D1 - eCFP exposure: 2000ms
CYB5D1::eCFP
poly glut. tubulin
merge
eCFP exposure: 2500ms
CYB5D1::eCFP
poly glut. tubulin
merge
#232 CCDC147 - eCFP exposure: 1000ms
CCDC147::eCFP
poly glut. tubulin
merge
eCFP exposure: 435ms
CCDC147::eCFP
poly glut. tubulin
merge
#257 C15orf26 - eCFP exposure: 807ms
C15orf26::eCFP
poly glut. tubulin
merge
eCFP exposure: 4000ms
C15orf26::eCFP
poly glut. tubulin
merge
#305 IQCA1 - eCFP exposure: 4000ms
IQCA1::eCFP
boosted gamma
merge
eCFP exposure: 4000ms
IQCA1::eCFP
boosted gamma
merge
#326 TEKT1 - eCFP exposure: 400ms
TEKT1::eCFP
poly glut. tubulin
merge
eCFP exposure: 6ms
TEKT1::eCFP
poly glut. tubulin
merge
#347 IPO5 - eCFP exposure: 333ms
IPO5::eCFP
poly glut. tubulin
merge
eCFP exposure: 1130ms
IPO5::eCFP
poly glut. tubulin
merge
#348 HSPA1L - eCFP exposure: 883ms
HSPA1L::eCFP
poly glut. tubulin
merge
eCFP exposure: 693ms
HSPA1L::eCFP
poly glut. tubulin
merge
