# Supplementary material for: CiliaCarta: An integrated and validated compendium of ciliary genes
Source: PLoS One. 2019 May 16;14(5):e0216705. doi: 10.1371/journal.pone.0216705 (PMC6522010; doi:10.1371/journal.pone.0216705)
Supplement: S5 Fig — a) OSCP-1 localization is not altered in mks-5(tm3100) worms with disrupted ciliary transition zones. Shown are phasmid cilia of worms expressing GFP-tagged OSCP-1 and XBX-1::tdTomato (ciliary marker). Scale bar; 5 μm. b) The ciliary transition zone is intact in oscp-1(gk699) mutant worms. Shown are images of phasmid cilia of worms expressing markers for transition zone proteins (MKS-5, NPHP-4 and MKS-2), the periciliary membrane (TRAM-1; normally excluded from the ciliary membrane), and the ciliary axoneme (DYF-11 and CHE-11). NPHP-4, MKS-2 and MKS-5 localizations are unaffected in oscp-1 worms, indicating that the composition of the transition zone is not dramatically affected. TRAM-1 remains excluded from the ciliary membrane of oscp-1 mutant indicating the membrane diffusion barrier at the transition zone membrane is intact. The ciliary axoneme markers (see also XBX-1 marker in the top panels) show that phasmid cilia are assembled and of grossly normal length in oscp-1 mutant worms. Scale bar; 5 μm. (PDF) [file pone.0216705.s005.pdf]

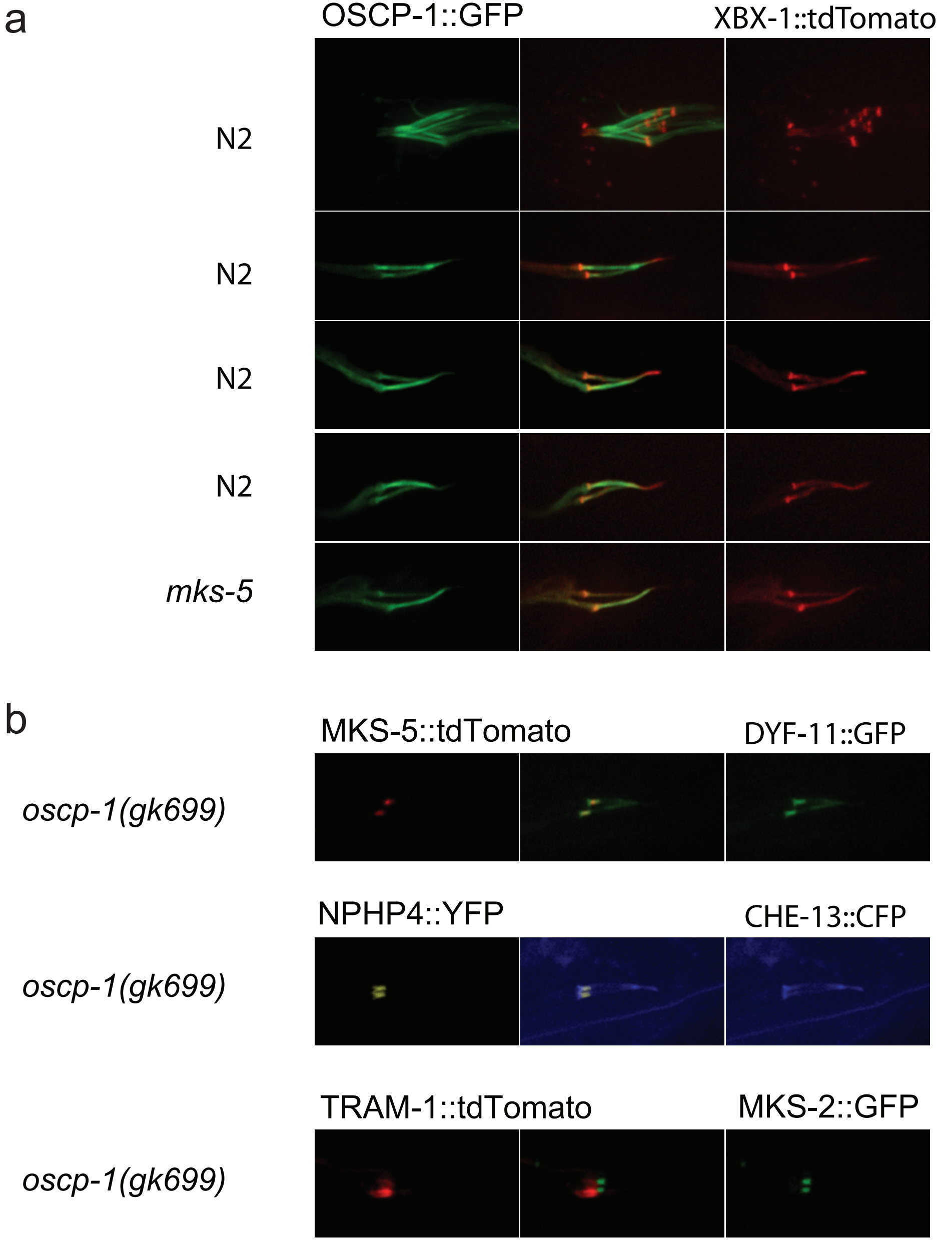
a
OSCP-1::GFP
XBX-1::tdTomato
N2
N2
N2
N2
mks-5
b
MKS-5::tdTomato
DYF-11::GFP
oscp-1(gk699)
NPHP4::YFP
CHE-13::CFP
oscp-1(gk699)
TRAM-1::tdTomato
MKS-2::GFP
oscp-1(gk699)
