# Supplementary material for: CiliaCarta: An integrated and validated compendium of ciliary genes
Source: PLoS One. 2019 May 16;14(5):e0216705. doi: 10.1371/journal.pone.0216705 (PMC6522010; doi:10.1371/journal.pone.0216705)
Supplement: S1 File — (DOCX) [file pone.0216705.s019.docx]

# Supplementary materials

## Bayesian classifier

### Performance and false discovery rate calculations

The performance of each data set for predicting ciliary genes as well as the integrated Bayesian classifier was evaluated using the positive and negative training sets. We determined the fraction, or recall, of the positive training set, which is also known as the sensitivity or true positive rate (TPR).


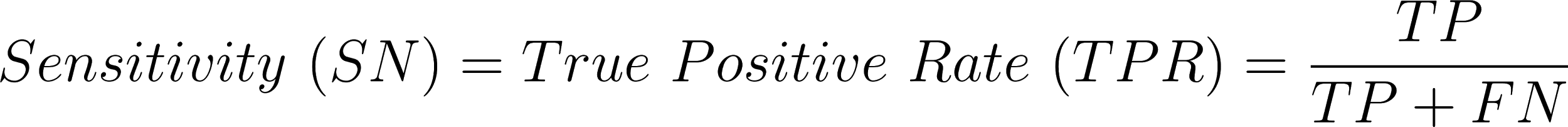


Where True Positives (TP) are true ciliary genes that are correctly discovered by the data set, and False Negatives (FN) are true ciliary genes that were not discovered by the data set. We also determined the fraction of the negative set retrieved by the data set, also known as the false positive rate (FPR).


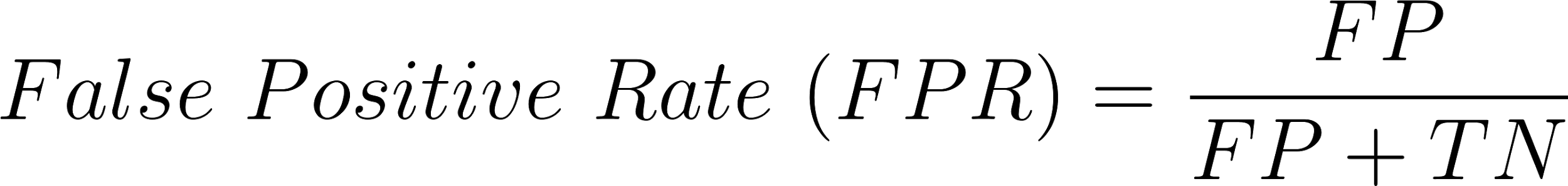


Where True Negatives (TN) are non-ciliary genes that are correctly excluded from the data set, and False Positives (FP) are non-ciliary genes incorrectly discovered in the data set. The FPR together with the TPR are used to calculate the predictive ability for each data set (see below).

The FPR is related to the specificity (SP), another well-known metric for the quality of a data set, as follows:


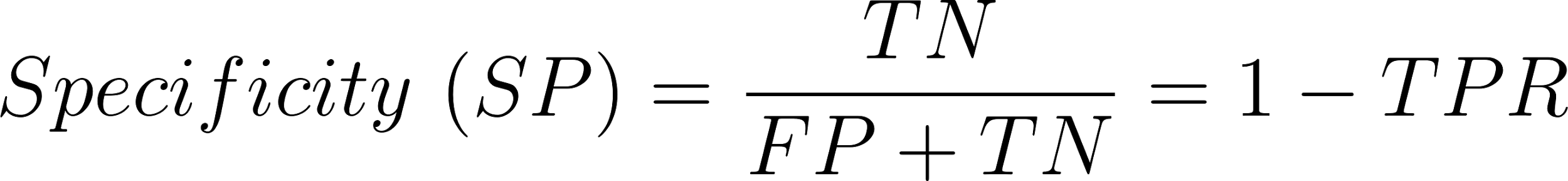


The false discovery rate (FDR) denotes the chance of encountering a false positive among a set of predictions and thus reflects the trustworthiness of a prediction. The FDR is given by:


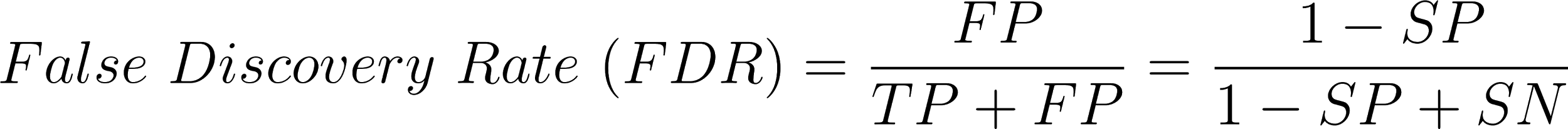


We use the FDR to determine the overall performance of the classifier given a specified threshold of the CiliaCarta Score. However, the FDR depends on both training sets and is sensitive to deviations in set size compared to the actual populations of positives and negatives for the whole genome, and thus we need to correct the canonical FDR equation for the differences in the population and set size:


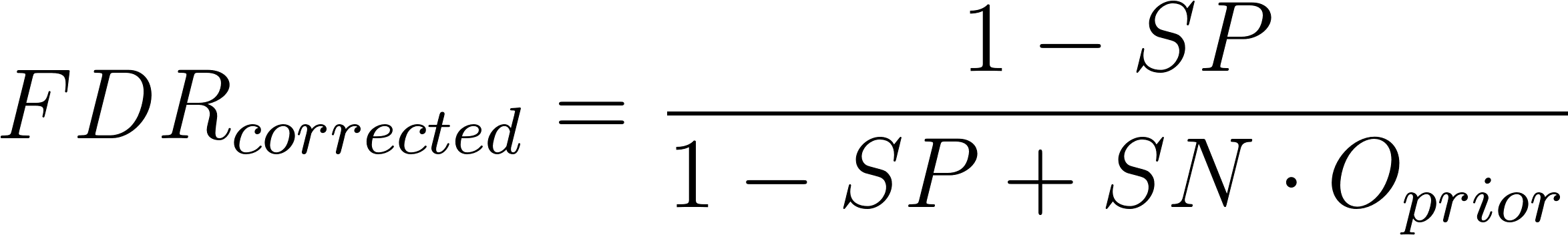


Throughout the text we refer to this adjusted FDR as the corrected FDR (cFDR). The positive predictive value (PPV) is directly related to the FDR and reflects the probability that a gene within the set threshold will indeed be ciliary:


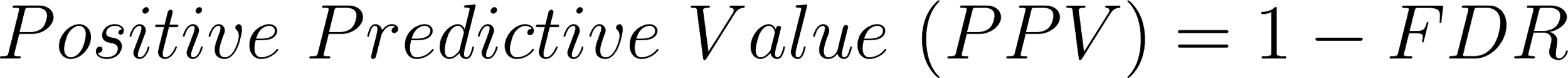


The PPV and FDR therefore reflect the chance of success or failure within a set of genes rather than for individual genes; i.e. they do not reflect the per gene probability for it being ciliary or not. Instead, the per-gene probabilities are reflected by the posterior odds, the CiliaCarta Score, see below.

At a cFDR cut-off of 25% our rank-ordered list of predictions covers 404 genes. However, this set contains genes from both the negative and the positive training sets, and after excluding those we obtain 285 predictions. This remaining set will however have a different FDR since we removed the training set genes. We obtain the FDR for the remaining 285 genes by: (i) calculating the number of TP and FP based on the cFDR threshold, (ii) subtracting the number of genes from the training sets (TP - genes in positive set, FP - genes in negative set) and (iii) recalculating the FDR using these adjusted TP and FP values. This results in a FDR of 33% for the 285 candidate predictions.

In our experimental validations 24 out of 36 genes are positive for ciliary phenotype and/or localization. We can derive an observed FDR directly from these numbers, i.e. 24 TP and 12 FP. The observed FDR is thus 12/36 = 33%, the same as the estimated FDR for our candidate genes.

### Calculation of the CiliaCarta Score using naive Bayesian integration

Naive Bayesian integration allows a direct comparison and weighing of many and diverse data sets describing the properties of ciliary genes and integrates these into a single probabilistic score for each gene accommodating for missing data(Jansen *et al*, 2003; Calvo *et al*, 2006; Tabach *et al*, 2013; van der Lee *et al*, 2015). This approach has been successfully used to predict for instance mitochondrial(Calvo *et al*, 2006) and innate immunity(van der Lee *et al*, 2015) genes.

For a given gene in the human genome we calculate the conditional probability that the gene is involved in ciliary processes given the observed evidence in the data sets. For our purposes, since we have only two possible outcomes (i.e. ciliary vs. non-ciliary) it is more convenient and appropriate to use odds instead. We can write the probability that a gene is ciliary given the outcome of the experiment in data set *i* (
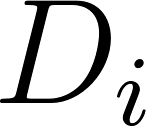
) for all data sets j:


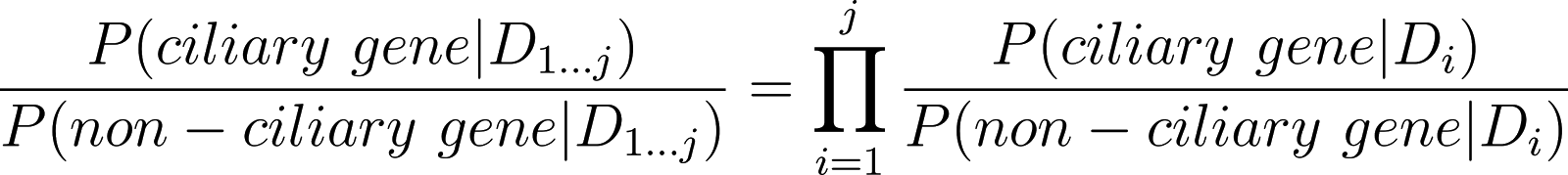


These odds cannot be calculated directly, but we can obtain them using Bayes’ theorem by approximating the reverse likelihood ratio *L* that a gene is observed in the data sets given it is either ciliary, or non-ciliary:


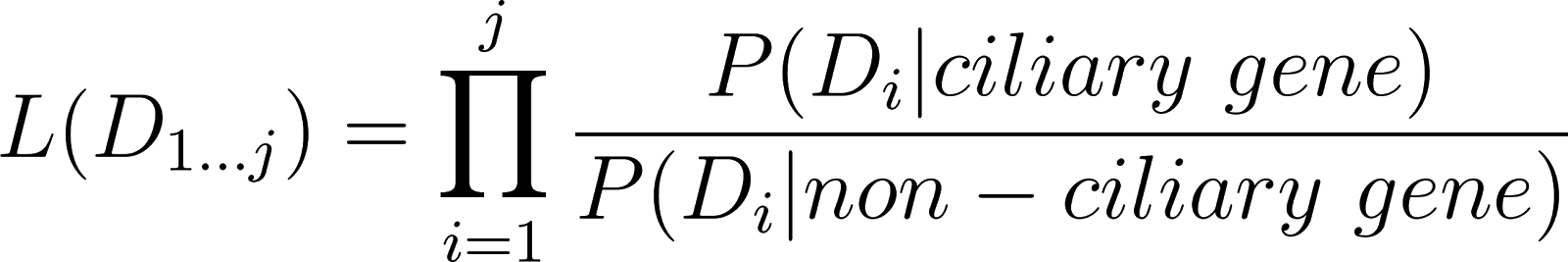


We can calculate this likelihood directly from the distribution of the training sets. This equates to the ratio between the data sets’ true positive rate for ciliary genes (i.e. the proportion of known ciliary genes retrieved), and the false positive rate for non-ciliary genes (i.e. the proportion of known non-ciliary genes retrieved). Using Bayes’ theorem we can now obtain the final (posterior) odds from the likelihood ratio *L* as follows:


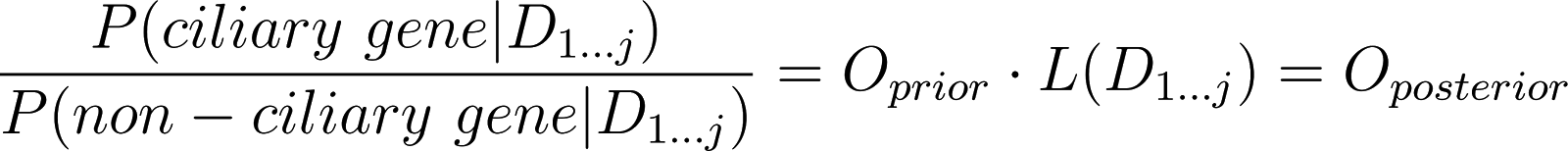


Where [
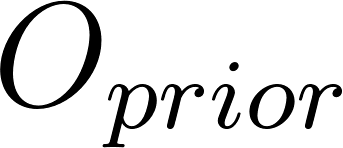
](http://www.codecogs.com/eqnedit.php?latex=O_%7Bprior%7D) is the prior odd, i.e. the odds for a gene to be ciliary if one would randomly sample from the genome:


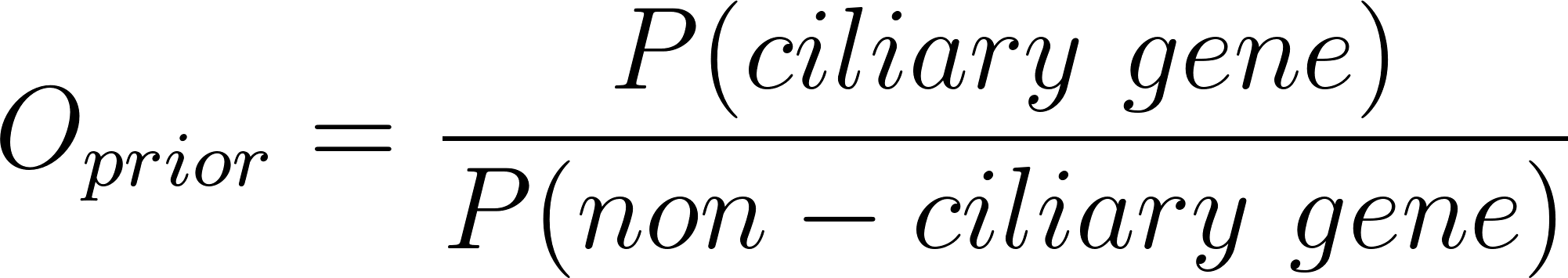


And thus we obtain the posterior probability for data sets *D_1…j_*:


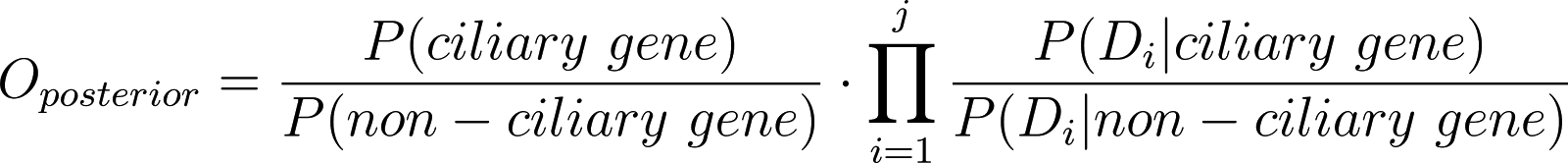


Finally we obtain the CiliaCarta Score by log_2_ transformation of the individual terms to get an additive score:


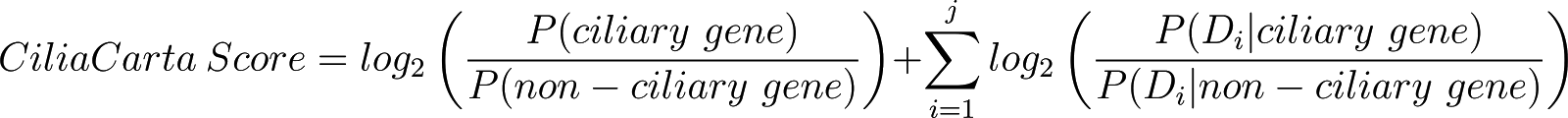


The additive nature of the log transformed posterior odds equation makes the contribution of each data set to the final score more insightful. It also makes the score robust against rounding errors otherwise encountered during multiplication of the untransformed odds. The log-odds for each individual data set per sub-category are listed in Supplementary table 7.

### Determining the prior

One of the elements in Bayesian calculations is estimating the prior: the *a priori* expectation of how many ciliary genes there are in the human genome. Although the ranking of genes does not depend on the prior, it is required to obtain a corrected false discovery rate (see above). Furthermore it gives a meaning to the posterior log odd scores (the CiliaCarta score), i.e. a positive log odd means that the gene is more likely to be ciliary than non-ciliary and a negative log odd means that it is more likely to be non-ciliary.

To our knowledge there is no substantiated estimate for the number of genes involved in the cilium. Currently 608 human proteins have been annotated as being part of the cilium in a combination of GO (GO:0005929 Cell Component Cilium & GO:0042384 Biological Process Cilium Assembly, Ensembl biomart as of December 3rd 2015) and the SCGS, but we can reasonably assume the total number of ciliary genes to be much higher. For instance, the ciliary proteome as identified by Liu *et al.* in the mouse photoreceptor sensory cilium entails 1185 to 1968 proteins, depending on the stringency of the filters applied(Liu *et al*, 2007). We have chosen a prior that we deem to be both reasonable and conservative: 5% of the human genome (i.e. 1135 ciliary genes). The
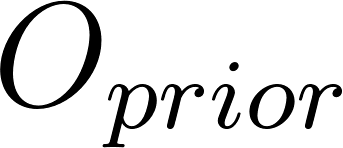
 then becomes:


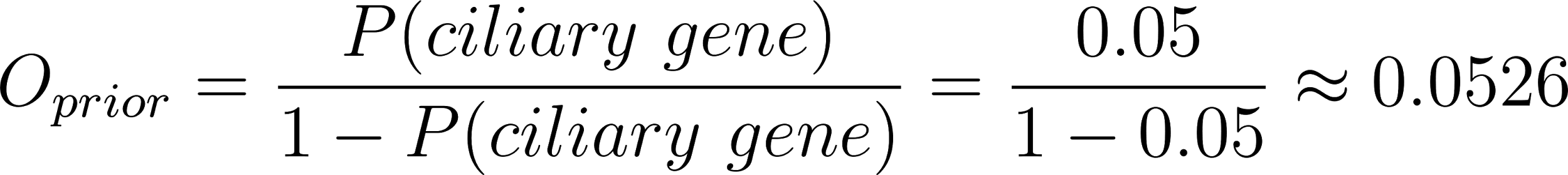


*Conditional independence*

An assumption of our naïve Bayesian approach is that the outcome of one data set is independent of the outcome of another. This assumption of independence is not always attainable, since for instance gene expression is to some extent biologically correlated to the presence of proteins in the proteomics data sets. Violations of the independence assumption can bias the predictions and can lead to an overestimation of the likelihood scores. However previous work has shown that, regardless of biological correlations between data sets, naive Bayesian integration of genomics data is highly effective to predict novel genes involved in a molecular system(Calvo *et al*, 2006; Tabach *et al*, 2013). Analysis of the correlations suggests that the data sets used to predict ciliary genes are largely complementary (Supplementary Fig. 10). Several data sets have high correlations, such as ciliary co-evolution and co-expression. However these data sets are methodologically and experimentally completely unrelated and thus the high correlation is purely based on the ability of the methods to predict ciliary genes.

### Ciliome size estimation

The Bayesian framework can be used to obtain a systematic estimate for the total number of ciliary genes in two ways. The first approach involves fitting a new
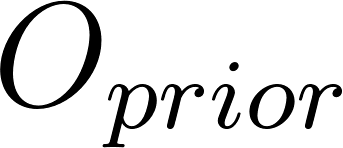
 based on the observed validation rate of our experiments; that is, we make use of the discrepancy between the expected and experimentally determined number of hits. We can estimate this new
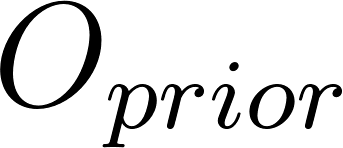
 by equating the c FDR of the Bayesian integration, which depends on our original
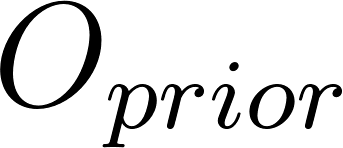
 to the observed FDR of the validation experiments:


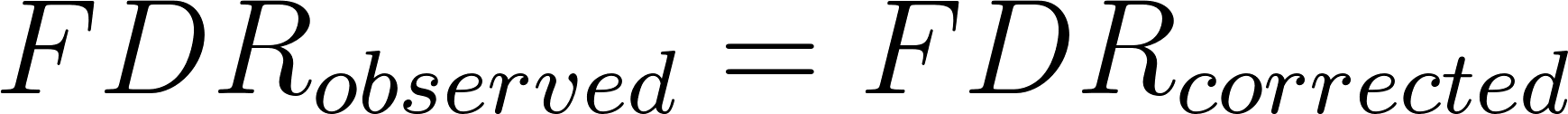


Where
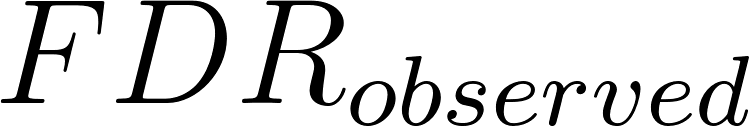
 is calculated from the TP and FP determined from the validation experiments (24 and 12 resp.). The
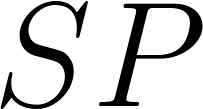
 and
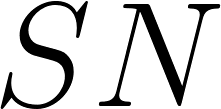
 for the cFDR can be obtained from the training sets. We then try to find an
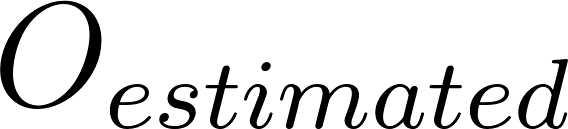
 for which the following equation holds:


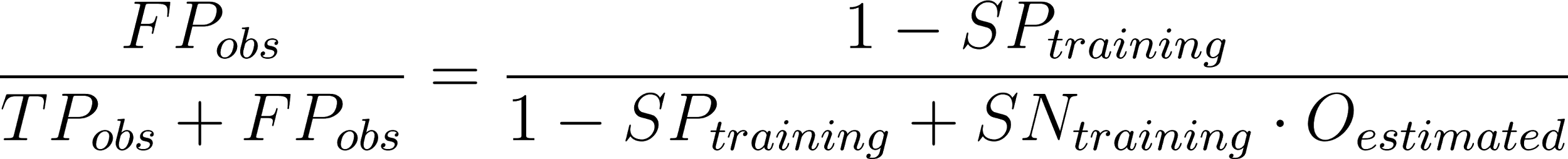


Atypically, in our study the observed FDR and the cFDR are equal (i.e. 33%), which indicates that
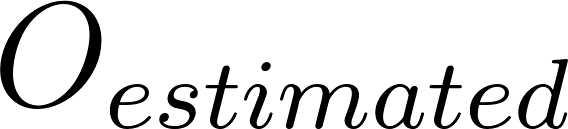
 essentially equals our initially chosen [
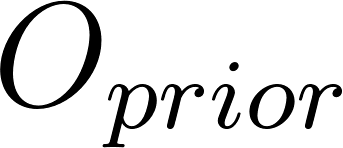
](http://www.codecogs.com/eqnedit.php?latex=O_%7Bprior%7D) (1135 ciliary genes).

The second approach to estimate the ciliome size is based on the Bayesian posterior probabilities obtained for each gene (the CiliaCarta Scores). To determine the expected value (i.e. the number of true positives) among a set of ciliary candidates, we considered each gene to be a random variable with a binary outcome (success, a true ciliary gene, or failure, not a ciliary gene) whose probability of success is defined by
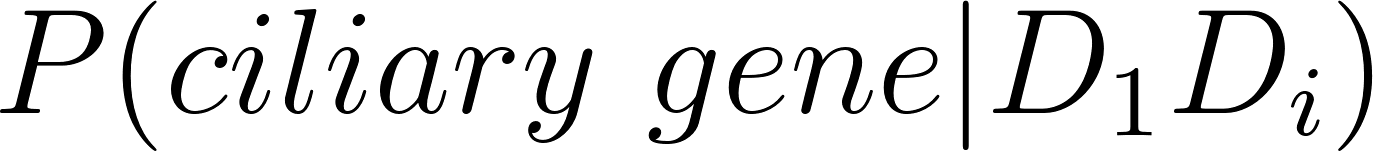
. Effectively this corresponds to a Bernoulli process with different probabilities for success or failure for each successive trial. Assuming independence, the expected value *E* for a set of *n* binary random variables (i.e. ciliary candidates) is equal to the sum of the expected values of the individual variables. The expected value for an individual binary random variable, in turn, equals its probability of success. From the posterior odds:


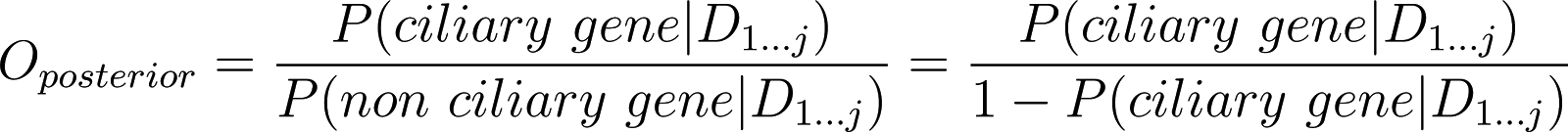


We can obtain the probability that a gene is ciliary by rewriting the above as:


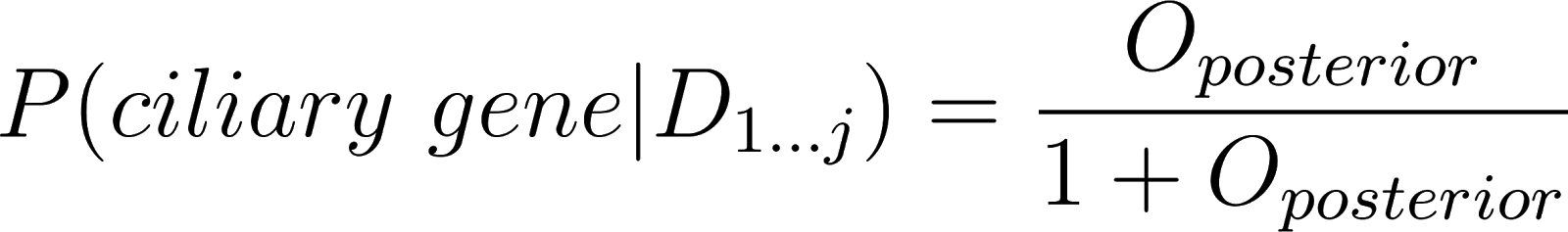


Since the CiliaCarta Score (CCS) is the log_2_ of the posterior odds we finally get:


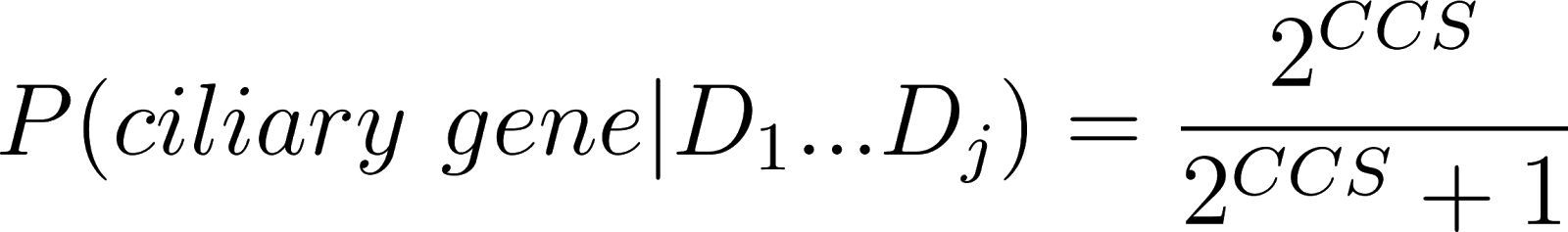


And thus to obtain the expected value E becomes:


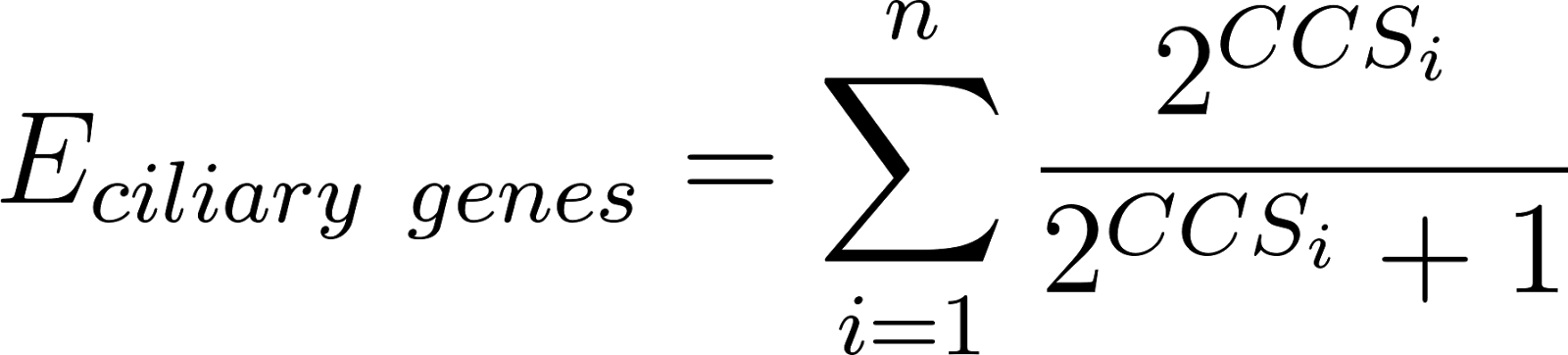


The expected value for the number of ciliary genes based on the Bayesian posterior probabilities was found to be 1273. It should be noted that the posterior CiliaCarta probabilities, and hence the expected value for a set of genes, depend on the prior estimation of the number of ciliary genes. We have above already established that our chosen prior was accurate based on our validation outcome. Indeed the posterior expected number of ciliary genes is close to the prior expected number of ciliary genes (1135, difference of 138). Averaging these two estimates we arrive at a total number of expected ciliary genes of approximately 1200 genes.

# References

Calvo S, Jain M, Xie X, Sheth SA, Chang B, Goldberger OA, Spinazzola A, Zeviani M, Carr SA & Mootha VK (2006) Systematic identification of human mitochondrial disease genes through integrative genomics. *Nat. Genet.* **38:** 576–82

Jansen R, Yu H, Greenbaum D, Kluger Y, Krogan NJ, Chung S, Emili A, Snyder M, Greenblatt JF & Gerstein M (2003) A Bayesian networks approach for predicting protein-protein interactions from genomic data. *Science* **302:** 449–53

van der Lee R, Feng Q, Langereis MA, ter Horst R, Szklarczyk R, Netea MG, Andeweg AC, van Kuppeveld FJM & Huynen MA (2015) Integrative Genomics-Based Discovery of Novel Regulators of the Innate Antiviral Response. *PLOS Comput. Biol.* **11:** e1004553

Liu Q, Tan G, Levenkova N, Li T, Pugh EN, Rux JJ, Speicher DW & Pierce EA (2007) The proteome of the mouse photoreceptor sensory cilium complex. *Mol. Cell. Proteomics* **6:** 1299–317

Tabach Y, Billi AC, Hayes GD, Newman MA, Zuk O, Gabel H, Kamath R, Yacoby K, Chapman B, Garcia SM, Borowsky M, Kim JK & Ruvkun G (2013) Identification of small RNA pathway genes using patterns of phylogenetic conservation and divergence. *Nature* **493:** 694–8
